# The requirement for cobalt in vitamin B_12_: A paradigm for protein metalation^☆^

**DOI:** 10.1016/j.bbamcr.2020.118896

**Published:** 2021-01

**Authors:** Deenah Osman, Anastasia Cooke, Tessa R. Young, Evelyne Deery, Nigel J. Robinson, Martin J. Warren

**Affiliations:** a Department of Biosciences, Durham University, Durham DH1 3LE, UK; b Department of Chemistry, Durham University, Durham DH1 3LE, UK; c School of Biosciences, University of Kent, Canterbury, Kent CT2 7NJ, UK; d Quadram Institute Bioscience, Norwich Research Park, Norwich NR4 7UQ, UK; e Biomedical Research Centre, University of East Anglia, Norwich NR4 7TJ, UK

**Keywords:** CoA, co-enzyme A, GDP, guanosine diphosphate, TCA, tricarboxylic acid, GC–MS, gas chromatography–mass spectrometry, ROS, reactive oxygen species, ABC, ATP-binding cassette, NiCoT, nickel/cobalt transporter, OM, outer membrane, ECF, Electron-coupled factor, SBP, substrate-binding protein, ATP, Adenosine triphosphate, MFS, major facilitator superfamily, RND, resistance nodulation division, AdoCbl, adenosylcobalamin, AqCbl, aquacobalamin, MeCbl, methylcobalamin, RBS, ribosome binding site, GTP, guanosine triphosphate, MCM, methylmalonyl-CoA mutase, Cobalamin, Cobamide, Metals, Chelation, Homeostasis, sensors

## Abstract

Vitamin B_12_, cobalamin, is a cobalt-containing ring-contracted modified tetrapyrrole that represents one of the most complex small molecules made by nature. In prokaryotes it is utilised as a cofactor, coenzyme, light sensor and gene regulator yet has a restricted role in assisting only two enzymes within specific eukaryotes including mammals. This deployment disparity is reflected in another unique attribute of vitamin B_12_ in that its biosynthesis is limited to only certain prokaryotes, with synthesisers pivotal in establishing mutualistic microbial communities. The core component of cobalamin is the corrin macrocycle that acts as the main ligand for the cobalt. Within this review we investigate why cobalt is paired specifically with the corrin ring, how cobalt is inserted during the biosynthetic process, how cobalt is made available within the cell and explore the cellular control of cobalt and cobalamin levels. The partitioning of cobalt for cobalamin biosynthesis exemplifies how cells assist metalation.

## Highlights

• The corrin-cobalt couple is ideal for forming metal-carbon bonds
• A fraction of bacteria make vitamin B_12_ and supply other organisms including humans
• The biogenesis of cobalamin: Cobalt chelatases and chaperones
• A thermodynamic framework for metalation has been established
• The partitioning of cobalt into cobalamin exemplifies how cells assist metalation

## 1 Vitamin B_12_ - its structure and biological roles

Vitamin B_12_ boasts a complex façade yet mediates an abundance of intricate chemistries that ultimately derive from the properties of a central cobalt ion. This essential dietary component first came to prominence a century ago when it was identified as the anti-pernicious anaemia factor that is present in raw liver [1,2]. The isolation of the nutrient from liver extracts was enhanced by the development of a microbial bioassay [3], using a bacterial vitamin B_12_ auxotroph, and culminated in the generation of purified bright red crystals [4,5], that were shown to contain cobalt [6,7], and which famously made their way into the hands of Dorothy Hodgkin for X-ray diffraction studies [8,9]. Through her pioneering work she was able to deduce the structure of vitamin B_12_ to reveal it as the most complex structure known at that time. It is an inauspicious coincidence that the name cobalt originates from German miners who when extracting the metal for its ability to colour glass believed their ores were contaminated by a pernicious goblin, or *kobold*, since when heated they emitted poisonous arsenic and sulphur containing gases – and that the main human cobalt deficiency in humans is related to vitamin B_12_ which is also associated with a pernicious ailment.

**Fig. 1:**
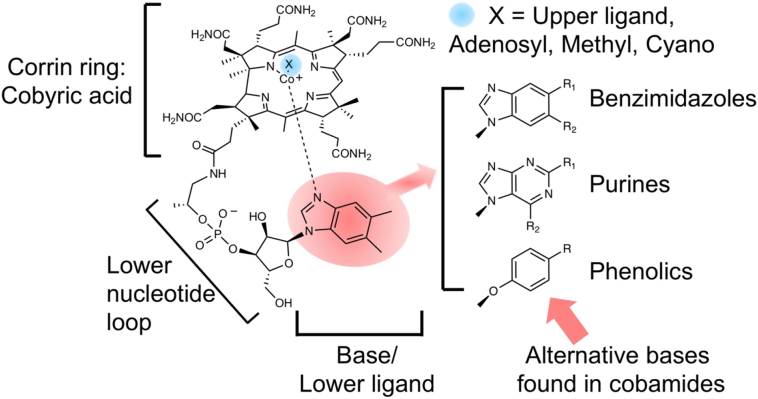
Structure of cobalamin (vitamin B_12_). The core component of cobalamin is the corrin ring, which houses a central cobalt ion. The corrin ring together with the cobalt is called cobyric acid. Attached to the propionic side chain of cobyric acid is a lower nucleotide loop, that contains an unusual base called dimethylbenzimidazole and which acts as a lower ligand to the cobalt ion. The upper ligand in vitamin B_12_, marked as a X in the diagram is a cyano group. In the biological forms of cobalamin, the upper ligand is usually a methyl or an adenosyl group. Some bacteria make variant forms of cobalamin where the dimethylbenzimidazole base is replaced with other bases such as other benzimidazoles (with variations around R1 and R2), purines (with variations around R1 and R2) and phenolics (with variations around R).

The structure of vitamin B_12_ can be considered in three parts (Fig. 1). Firstly, there is a modified tetrapyrrole that ligands a central cobalt ion. This tetrapyrrole-derived ring is unusual in that it has undergone a ring-contraction process meaning that one of the bridging (*meso*) carbon atoms that are used to connect the four pyrrole rings has been eliminated, thereby generating a macrocycle that is both contracted and lop-sided in comparison to the tetrapyrrole-frameworks that are associated with heme and chlorophylls [10]. This contracted ring structure is called a corrin. Secondly, the molecule contains a nucleotide loop that houses an unusual base, which in vitamin B_12_ is called dimethylbenzimidazole. The nucleotide loop is attached to one of the propionate side chains of the corrin ring through an aminopropanol linker, extending underneath the plane of the corrin ring such that the dimethylbenzimidazole base is able to act as a lower ligand for the cobalt. Finally, the third component of vitamin is the upper ligand to the cobalt ion, which in vitamin B_12_ is cyanide. The corrin ring and the lower nucleotide loop with its dimethylbenzimidazole base represents a molecule that is called cobalamin. Technically, vitamin B_12_ is therefore cyanocobalamin but vitamin B_12_ is quite often used loosely to refer to cobalamin. The cyano group in vitamin B_12_ is a consequence of the way the molecule is isolated, where cyanide is added to help its extraction and purification [11].

Some bacteria make alternative forms of cobalamin that differ in the nature of the lower nucleotide loop (Fig. 1). Here, the differences largely relate to the base that is incorporated into the loop so, for instance, some bacteria incorporate adenine rather than dimethylbenzimidazole to give rise to a cobalamin analogue that is referred to as pseudocobalamin [12]. However, across different bacterial species around 15 different variants of cobalamin are made and these are collectively referred to as cobamides or corrinoids [13,14]. Cobalamin therefore is just one member of a broader cobamide family that all contain the same corrin ring with a liganded cobalt ion. It is the relationship between the cobalt ion and the corrin ring that is important with respect to its biological function [15,16].

The main active biological forms of cobalamin are where the upper ligand is either a methyl or an adenosyl group, which are present in methylcobalamin and adenosylcobalamin, respectively [17,18]. Methylcobalamin acts as a cofactor in a number of methyl transferase reactions, including in methionine synthase. Adenosylcobalamin acts as a coenzyme in rearrangement/isomerase reactions such as with methylmalonyl CoA mutase. These two enzymes catalyse probably the two best-known B_12_ -dependent reactions which also represent the only two known B_12_ -dependent processes in mammals. However, in prokaryotes there are a range of other cobamide-dependent enzymes including the diol dehydratases, ethanolamine ammonia lyase, ribonucleotide reductase, reductive dehalogenases and a host of radical SAM enzymes [13,19]. Adenosylcobalamin has also been shown to act as a light sensor in the CarH transcription factor [20]. Finally, cobamides can also directly influence levels of transcription and translation through interacting with riboswitches, where binding of specific cobamide forms to regulatory regions within the mRNA controls the production of encoded proteins (Section 5.3.1) [21,22]. Most cobamide riboswitches regulate genes associated with cobalt transport and cobalamin metabolism.

### 1.1 Chemistry of cobalt and the corrin ring

**Fig. 2:**
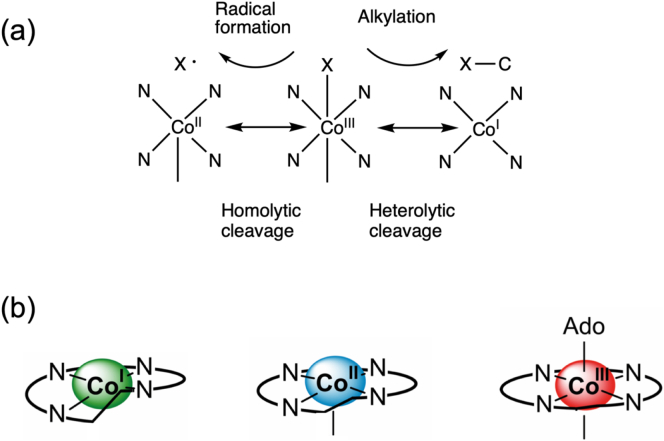
Cobalt coordination and chemistry associated with cobamides. (a) Co(III) corrinoids such as adenosylcobalamin (where X = deoxyadenosyl) can undergo homolytic cleavage to generate a Co(II) species and an adenosyl radical (X•), whereas methylcobalamin undergoes heterolytic cleavage to generate a Co(I) species and a methylated product. (b) The corrin ring naturally adopts a helical arrangement and this fits well with Co(I). More increasingly planar forms of the corrin ring are generated with Co(II) and Co(I) respectively. The ability to transition between planar and helical conformations means that the corrin ring acts as an entatic state module.

One of the long-standing questions concerning cobamides relates to why nature has selected the combination of cobalt and the corrin ring as a metalloprosthetic group partnership. What is the advantage of a corrin ring over the versatile porphyrin macrocycle of heme, and what is it about cobalt that so suits it for B_12_-dependent processes? This is particularly relevant given that (i) the availability of cobalt as a trace element varies significantly on both land and in water [[23], [24], [25]], and (ii) that the corrin ring is so difficult to make [[26], [27], [28]]. The main driving force for utilising cobalt is the chemistry that is mediated by the metal. Fundamental to this is the ability of cobalt to form metal-carbon bonds, the formation of which is facilitated by the powerful nucleophilicity of the Co(I) species [17,18,29]. The ability to control the nature of the Co-carbon complex, especially with control over homo- or heterolytic cleavage of this bond is crucial to why nature has selected the cobalt-corrin couple (Fig. 2a) [30,31].

For instance, in the methyltransfer reaction associated with methionine synthase the cobalt in methylcobamide is in the Co(III) state and the methyl group is transferred after heterolytic cleavage of the Co-CH_3_ bond to generate the nucleophilic Co(I) species, which rapidly acquires another methyl group from Me-tetrahydrofolate [17,18]. In this process the cobamide cycles between Co(III) and Co(I) allowing for efficient transfer of the methyl group from methyl-tetrahydrofolate to homocysteine in the generation of methionine with the cobamide acting as a recycling cofactor. With isomerisation reactions the Co-carbon bond of adenosylcobamide undergoes homolytic cleavage, converting the Co(III) of adenosylcobamide to a Co(II) species and generating an adenosyl radical. The adenosyl radical is then able to abstract a hydrogen from the substrate, thereby inducing substrate radical formation and rearrangement, prior to hydrogen abstraction from the adenosyl group and reformation of the Co(III) adenosylcobamide [17,18]. The chemistry of cobamide-dependent reactions therefore relates to the controlled change in the oxidation state of the cobalt ion, changes that also reflect preferences for specific co-ordination of the metal ion. Thus, within the corrin macrocycle Co(III) prefers to bind in a 6 coordinate fashion, Co(II) in 5 coordinate fashion and Co(I) in a 4 coordinate manner. These changes in the oxidation state of the cobalt ion are, in part, facilitated by the corrin ring, which unlike like the planar ring of the porphyrin adopts a helical conformation [15]. The liganding of the cobalt ion into the corrin ring induces changes in the level of helicity within the corrin structure depending upon the oxidation state of the metal such that the corrin ring acts as an entatic state module that facilitates metal-oxidative state conversion (Fig. 2b) [15,32].

## 2 Vitamin B_12_ in prokaryotes and eukaryotes

The inclusion of both upper and lower ligands for cobalt within the corrin framework make cobamides much more complex structures than observed with other modified tetrapyrroles [10]. This complexity is reflected in equally convoluted biosynthetic pathways, involving upwards of 30 enzymatic steps for their complete *de novo* biogenesis [33]. This molecular intricacy is the likely explanation as to why the biosynthesis of cobamides has remained a prokaryotic prerogative, with the synthesis never making the transition to eukaryotes [26]. Even within the prokaryotic world there is a significant amount of variation as to which bacteria make the nutrient and those that cannot make it but require it. It appears that somewhere around 90% of bacteria contain a B_12_-dependent enzyme, yet only around a third of bacteria have the ability to make the nutrient [34]. This discrepancy between the synthesisers and utilisers likely reflects an attempt to share metabolic burden between microbes that occupy similar environmental niches and participate in mutualistic interactions [34,35].

Cobamides are very efficient cofactors and coenzymes, and coupled with the fact that they are very stable entities means that, in general, only very small quantities of the nutrient are required within a cell. For instance, in humans the dietary requirement for cobalamin is around 2 μg per day and hence a small amount of vitamin B_12_ goes a long way [36]. However, many systems have evolved to live in a B_12_-less setting. Higher plants, fungi and yeasts all exist in the absence of cobamides, with cobamide-dependent enzymes replaced with less efficient alternatives. This has implications for the food chain since crop-grazing mammals have to acquire their dietary requirement for cobalamin from alternative sources. For ruminants this demand for the nutrient is met by the ruminal microbiota that produce the necessary cobalamin, which is then absorbed into the circulatory system. Mono-gastric crop-feeding animals acquire their cobalamin either from surface bacteria on their food or by coprophagy. Humans acquire their cobalamin generally from dairy and meat produce. Although the human gastro-intestinal microbiome produces a range of cobamides, cobalamin only represents a minor component of this total [37]. Moreover, the human cobalamin uptake mechanism is located within the small intestine [38] whereas the gut microbiota is largely located within the large intestine. The take home message from this is that humans on vegetarian and more specifically vegan diets are prone to B_12_-deficiency [36].

## 3 Biosynthesis of vitamin B_12_

**Fig. 3:**
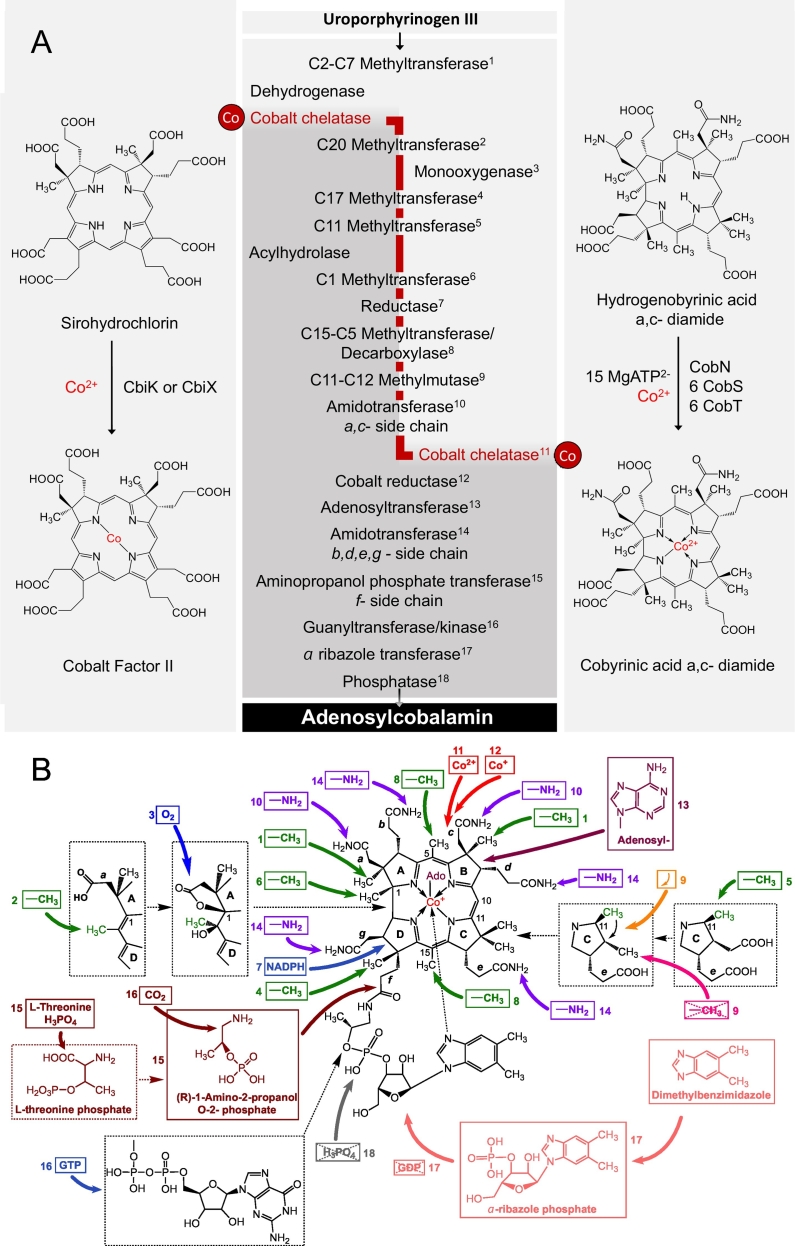
The biogenesis of cobalamin. (a) The biosynthesis of cobalamin from the common tetrapyrrole primogenitor uroporphyrinogen III involves in excess of twenty steps. Although there are two pathways for the synthesis of cobalamin, referred to as the aerobic (or cobalt-late) and anaerobic (cobalt early) routes, the series of methylation, amidation and rearrangement reactions are broadly similar and these steps are highlighted. The cobalt-insertion steps are also shown, with the cobalt-early insertion shown on the left hand side and the cobalt-late insertion stage shown on the right hand side. (b) The modifications that are associated with the cobalt-late pathway are highlighted and cross-referenced with (a). The Figure highlights the extensive modification that take place during the biogenesis of cobalamin.

As alluded to earlier, the biogenesis of cobamides such as cobalamin is a complex process, and as with all modified tetrapyrroles the construction is based on the primogenitor blueprint of uroporphyrinogen III [10]. The transformation of uroporphyrinogen III into the corrin ring component of cobalamin, a molecule called cobyric acid, requires 8 methylations, 6 amidations, extrusion of the C20 *meso* position, decarboxylation of an acetic acid side chain and the insertion of cobalt (Fig. 3). The conversion of cobyric acid into cobalamin requires the attachment of a threonine-derived aminopropanol linker to the remaining propionic acid side chain to give cobinamide, activation of cobinamide with GDP and then substitution of the GDP moiety with α-ribazole-5′-P. The α-ribazole-5′-P is synthesised by attachment of the ribosyl-5′-phosphate moiety of nicotinate mononucleotide to dimethylbenzimidazole (Fig. 3b). More detailed reviews of cobalamin biosynthesis can be found elsewhere [13,33,39,40].

The biosynthesis of cobamides is further confounded by the presence of two genetically distinct but similar biosynthetic pathways for the construction of the corrin ring component of the molecule [10,41]. These pathways differ in their requirement for molecular oxygen and the timing of cobalt insertion (Fig. 3a). In the anaerobic pathway cobalt is inserted at an early stage, prior to ring contraction, and the pathway is independent of molecular oxygen. By way of contrast the aerobic pathway requires molecular oxygen to help promote the contraction process and inserts cobalt after all the methylations have taken place. Although these two routes are referred to as the anaerobic and aerobic pathways they should more accurately be called the early or late cobalt insertion pathways, respectively [27]. Despite the differences in the timing of cobalt insertion and the mechanism for ring contraction many of the other enzymes that are associated with methylations and amidations are very similar and operate in the same order along the pathway. The two pathways start from uroporphyrinogen III and progress *via* an ordered attachment of methylation and amidation reactions interspersed with ring contraction, decarboxylation, methyl migration and adenosylation reactions. The two pathways diverge after the first two methylation events, facilitated by the uroporphyrinogen III methyltransferase, and re-join around the point of the final amidations.

## 4 Cobalt toxicity

Cobalt deficiency is a challenge for those organisms which synthesise B_12_, while all organisms must handle this metal carefully since cobalt can mediate a range of adverse effects. Metallostasis for cobalt, like other transition metals, is necessary at the upper-end of the tolerable range to avoid toxicity arising from aberrant reactivity including mismetalation. Cobalt is a well-known cause of contact dermatitis, leading to it being named ‘Allergen of the Year’ in 2016 by the American Contact Dermatitis Society. Severe cases of cobalt toxicity in humans can cause neurotoxicity, pneumonia and increase lung cancer risk when inhaled [42]. In bacteria, cobalt toxicity arises due to the disruption of iron-sulphur cluster formation, mismetalation of metallocomplexes and the formation of reactive oxygen species.

### 4.1 Limitation of iron availability

Many of the mechanisms of cobalt toxicity are due to competition between iron and cobalt. Iron and cobalt are both similar in size and charge. One of the effects of cobalt stress is the reduction of intracellular iron content by 50% [43]. The addition of exogenous iron has been shown to restore bacterial growth at high concentrations of cobalt, suggesting that cobalt can out compete iron at metal binding sites. Cobalt has been shown to compete with the metal binding site of Fur, the iron regulatory protein, and significantly impair its activity [44]. Fur binds iron as a corepressor, so iron availability mediates the activity of Fur. In *E. coli* Fur controls the iron-dependent expression over 90 genes, including genes encoding iron uptake and enzymes involved in glycolysis and the TCA cycle [45]. The notion of iron and cobalt competition is best shown when Fur is deleted from *E. coli*. These strains have unregulated iron import and are far less sensitive to cobalt than wildtype strains [46,47].

### 4.2 Iron-sulphur clusters

Fe—S clusters are most commonly found in either the [4Fe—4S], [3Fe—4S] or [2Fe—2S] forms. So far there have been three biosynthetic pathways identified in *E. coli*: the ISC, SUF and CSD. These pathways are induced at different times. The ISC pathway is thought to be used under normal growth whereas the SUF is believed to be used under periods of cell stress that result in Fe—S cluster degradation [48,49]. The biological role and timing of the CSD pathway is still unknown. The formation of Fe—S clusters in the ISC and SUF pathways requires protein scaffolds IscU, IscA and SufA [[50], [51], [52]]. These are important in the formation and delivery of clusters to the target apoprotein. The exposure of *E. coli* to 200 μM CoCl_2_ resulted in the cobalt and iron competing with scaffold proteins IscA and SufA and the insertion of mixed [Fe/Co-S] clusters into proteins [43]. Mature iron-sulphur clusters have been shown not to be affected by cobalt. MiaB, a [4Fe—4S] radical SAM enzyme involved in TRNA modification; FhuF, a [2Fe—2S] enzyme involved in ferrisiderophore reduction and aconitase were incubated with CoCl_2_ *in vitro* and had little effect upon activity [43].

Many Fe—S proteins are found within the tricarboxylic acid (TCA) cycle. Aconitase catalyses the conversion of citrate to isocitrate in the TCA cycle and an Fe—S cluster. It has been previously shown that presence of toxic levels of cobalt during the growth of *E. coli* caused the activity of aconitase to drop 70–80% [43]. Succinic dehydrogenase and fumarate reductase also contain Fe—S clusters and likely cease functioning due to toxic levels of cobalt, causing metabolic dead ends. Majtan et al. showed that *E. coli* adapts to oxidative stress by utilising modified mixed acid fermentation under aerobic conditions [53]. High levels of citrate, 2-hydroxyglutarate, succinate, lactate, fumarate and malate were found in cobalt-containing growth media through GC/MS analysis [53]. These intermediates correlate with TCA enzymes aconitase, succinic dehydrogenase and fumarate reductases placement within the cycle.

### 4.3 Oxidative stress and DNA damage

Cobalt-induced formation of reactive oxygen species (ROS) has been well documented in both eukaryotic and prokaryotic cells due to its ability to form different oxidative states. It has been shown *in vitro* that cobalt in reduced states can react with O_2_ and H_2_O_2_ through Fenton reactions to generate ROS, superoxides and hydroxy radicals that can go on to damage DNA and inhibit DNA repair mechanisms [54].

It has been observed that *E. coli* grown in the presence of 250 μM CoCl_2_ has a 1.4-fold increase in ROS when measured using an oxidative stress-sensitive fluorescent probe [46]. Conversely to other studies, Kumar et al. found that the addition of 500 μM CoCl_2_ to *E. coli* generated no ROS species and that DNA was damaged through an oxidative stress-independent pathway [47]. The addition of nickel and cobalt was demonstrated to inhibit the rate of DNA replication up to 50%. RecBCD, a helicase that initiates the SOS response, was inhibited, leading to DNA being damaged without an SOS response. Further to these findings, it was also noted that the generation of mixed acids, as described earlier, in combination with an acidic environment only enhanced cobalt toxicity.

### 4.4 Interference in sulphur assimilation

Thorgersen and Downs found that cobalt may affect sulphur assimilation in *Salmonella enterica* serovar Typhimurium (hereafter *Salmonella*) [44]. The sulphur assimilation pathway uses CysJI to convert sulphites to sulphides, which are then converted to cysteine. CysJI contains a [4Fe—4S] site and requires siroheme as a cofactor. It was shown there was a correlation between high exogenous cobalt concentrations and minimal CysJI activity. Both siroheme and [4Fe—4S] require iron – an element whose availability is reduced during cobalt toxicity. Cysteine is also the source of sulphur during [Fe—S] cluster formation, meaning cobalt affects both components of the complex.

### 4.5 Mismetalation of tetrapyrroles

Cobalt and iron have been shown to compete again during the formation of heme in *E. coli*, where protoporphyrin IX is mismetalated with cobalt [53]. Cobalt-protoprophyrin IX was found inside membrane-bound cytochromes, leading to the inhibition of the electron transport chain and decreased respiration. This is not the first account of the ferrochelatase inserting incorrect metals. The formation of zinc-protoporphyrin is well document and arises during periods of iron limitation [55].

## 5 The components of cobalt homeostasis

Metallostasis for cobalt, in common with other metals, involves the combined actions of, for example, importers and exporters (Section 5.1), delivery proteins (Section 5.4) and sensors that match supply with demand while avoiding toxic excess (Section 5.2) [56,57]. Peculiar to cobalt metallostasis in some bacteria is the use of B_12_-riboswitches to coordinate the substantial contribution of vitamin B_12_ biosynthesis to cobalt demand. Riboswitches also integrate B_12_ uptake with demand when the cofactor itself is available (Section 5.3).

### 5.1 Transporters

#### 5.1.1 Cobalt uptake

**Fig. 4:**
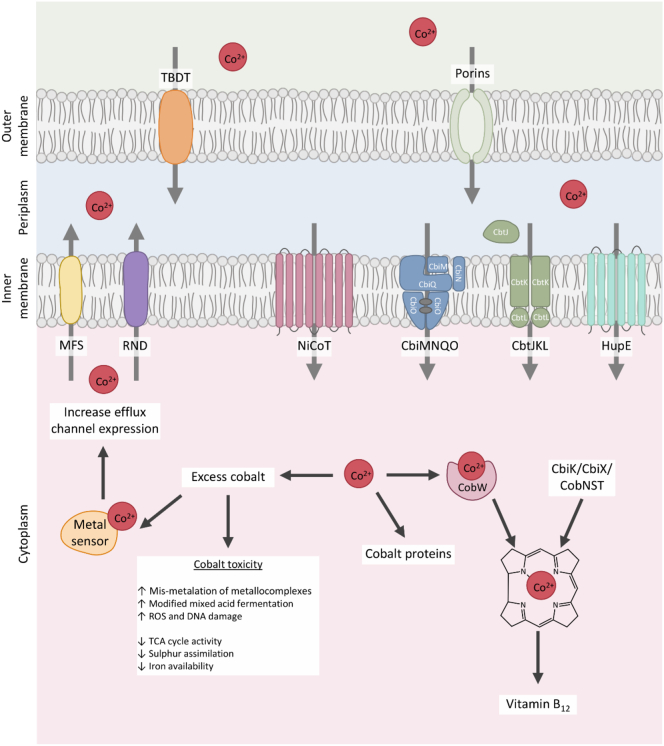
Cobalt transporters. Cobalt is transported through the outer membrane by porins or by TonB-dependent transporters (TBDT). From the periplasm, cobalt is transported through the inner membrane by a range of primary and secondary transporters. When cobalt is in excess, cobalt is transported out of the cytoplasm by MFS (major facilitator) permeases and RND (resistance nodulation division pumps) whose expression is controlled by a variety of metal-sensing proteins.

As there are only a handful of non-corrin cobalt-containing enzymes, cobalt uptake and requirement is generally distributed among organisms that can synthesise vitamin B_12_ *de novo*. Bacteria have developed many uptake systems for cobalt that sustain growth without inducing cobalt toxicity (Fig. 4). These transporters are largely highly specific but low capacity due to the toxic nature of cobalt. Many now-known cobalt transporters were identified *in silica* due to their location within B_12_ biosynthetic operons or their presence elsewhere in the genome under the control of an adenosylcobalamin riboswitch. Cobalt is primarily transported by ATP-binding cassette (ABC) transport systems with the addition of secondary transport systems such as nickel-cobalt (NiCoT) permeases (Table 1) .

**Table 1:** Transport proteins involved in cobalt homeostasis.

Transporter	Transporter family	Organism	Metal specificity	Reference
Cobalt uptake				
CbtJKL	ABC	*Sinorhizobium meliloti*	Co(II)	[61]
CbiMNQO	ECF	*Salmonella*	Co(II), Ni(II)	[22]
NhlF	NiCoT	*Rhodococcus rhodochorus*	Co(II), Ni(II)	[70,71]
HupE	HupE/UreJ	*Synechocystis sp.* PCC 6803	Co(II)	[73]
CorA	CorA	*Escherichia coli*	Mg(II), Co(II), Ni(II)	[238,239]
		*Thermotoga maritima*	Co(II)^,^ Mg(II)	[77]

Cobalt sensing and efflux				
RcnA	MFS	*Escherichia coli*	Ni(II), Co(II)	[240,241]
CoaT		*Synechocystis sp.* PCC 6803	Co(II)	[94]
CzcABC	RND	*Cupriavidus mtallidurans* CH34	Zn(II), Co(II), Cd^II^	[242]
CnrABC			Co(II), Ni(II)	[243]

ABC – ATP-binding cassette, ECF – electron-coupled factor, NiCoT – nickel/cobalt transporter, MFS – major facilitator factor, RND – resistance nodulation division.

##### 5.1.1.1 Transport across the outer membrane

In Gram-negative bacteria, cobalt must cross both the inner and outer membranes. Large molecules, such as vitamin B_12_, are transported across the membrane by TonB-dependent transport. In the case of vitamin B_12_, the outer membrane receptor BtuB transports B_12_ into the periplasm [58]. Some TonB OM receptors have been identified and predicted to be cobalt-specific. In the case of *Novosphinogobium aromaticivorans*, the gene encoding a TonB outer membrane receptor is preceded by an adenosylcobalamin riboswitch and then followed by genes encoding a NiCoT permease and cobaltochelatase, CobW (Section 5.4) [22]. No cobalt-specific outer membrane receptors have been confirmed so far but some have been identified for nickel scavenging in *Helicobacter* species. In *H. pylori,* the outer membrane receptor FrpB4 and the ExbB/ExbD/TonB complex were required for efficient nickel scavenging [59]. A similar protein, NikH, was identified in *H. mustelae* [60].

##### 5.1.1.2 Canonical ABC transporters

An ABC-type cobalt importer, CbtJKL, has been reported in *Sinorhizobium meliloti* [61]. Originally thought to encode a cobalamin transporter due to sequence similarity between CbtJ and BtuF, the CbtJKL complex transports Co(II) through the inner membrane into the cytoplasm. The complex was found to be responsible for cobalt transport when *S. meliloti* strains with a *cbtJKL* knock-out could only be grown in the presence of 1–10 μM CoCl_2_. It has not been reported on whether this system can also transport nickel in addition to cobalt. *cbtJKL* is found on the pSymB megaplasmid of *S. meliloti* and is unsurprisingly under the control of an adenosylcobalamin riboswitch [61].

##### 5.1.1.3 ECF transporters

Electron-coupled factor (ECF) transporters are a subfamily of the ABC transporter family that lack the cytoplasmic substrate-binding protein (SBP) found in ABC transporters. Cobalt-specific ECF transporters were first identified in *Salmonella* where a cluster of four genes, *cbiMNQO*, were found in the B_12_ biosynthetic operon [62]. This was later shown to be a cobalt-specific transport system [22]. ECF transporters consist of a substrate-specific transmembrane domain (S unit), a transmembrane protein (T unit) and two ABC ATPase domains (A unit) [63].

CbiMNQO contains a T unit (CbiQ), an ABC ATPase (CbiO) and two S unit transmembrane proteins (CbiMN). CbiMQO form a stable complex with a stoichiometry of 1:1:2 and a crystal structure of the complex was published in 2017 [64]. CbiM lies across the periplasmic face of the inner membrane with CbiQ wrapping around in a ‘C' shape. Two CbiO units are found beneath CbiQ and CbiM, forming a cone shape and the substrate binding site is a large cleft formed by CbiQ and CbiO. The interactions of CbiN and the CbiMQO complex are weak: it has been frequently demonstrated that CbiN cannot be co-purified with the rest of the complex and only interacts transiently, but CbiM and CbiN are the minimal required proteins to transport cobalt across the membrane [22,[64], [65], [66]]. CbiN has a short flexible extracytoplasmic loop between its two transmembrane helices that lies on top of the metal binding pocket of CbiM. It has been shown that truncations or modifications to this loop severely impair cobalt transport though the mechanism behind their interactions is still not well understood [67]. It is understood that CbiM provides ATPase activity to the complex without the presence of CbiN, and that CbiO units undergo ATP binding and product release-induced conformational change, but the full transport cycle is yet to be elucidated [65].

##### 5.1.1.4 NiCoT permeases

The most diverse group of cobalt transporters are the nickel/cobalt permeases (NiCoT; TC2.A52). NiCoTs are found across many bacteria and some archaea and urease-producing fungi [22,68]. NiCoTs have 6–8 transmembrane helices containing 4 characteristic domains, show in Table 2, of which Domain 2 has been demonstrated to be responsible for metal binding [69]. The N- and C-terminal helices are separated by a large charged and hydrophilic loop. The first cobalt-specific NiCoT identified was NhlF from *Rhodococcus rhodochorus*, but later shown to also transport nickel [70,71]. NiCoTs can be divided into three classes based on their substrate specificity: (i) Class I only transport nickel; (ii) Class II transport nickel and cobalt with preference for cobalt, *e.g.* NhlF; and (iii) Class III transport nickel and cobalt with a preference for nickel. The substrate specificity of this family cannot be predicted by sequence identity. The best predictor of substrate specificity is genomic localisation. Many genes encoding nickel-specific NiCoTs are located downstream of [NiFe] hydrogenase operons or under transcriptional control by NikR, whereas genes encoding predicted cobalt-transporting NiCoTs are located within B_12_ biosynthetic operons, or close to cobalt-containing nitrile hydratase genes [72].

**Table 2:** Signature domains of nickel-cobalt permeases. From [244].

Domain	Motif
TM2	(R/K)HAXDADH(I/L)
TM3	FXXGHS(T/S)(V/I)V
TM5	LGX(D/E)T(A/S)(T/S)E
TM6	GMXXXD(T/S)XD

##### 5.1.1.5 HupE/UreJ

Members of the HupE/UreJ transporter family are distributed throughout proteobacteria and cyanobacteria. Many are found within [NiFe] hydrogenase and urease gene clusters, leading to their assumed function in nickel uptake. However, some homologs in cyanobacteria have been identified as cobalt transporters as they are regulated by an adenosylcobalamin riboswitch [21,22]. HupE cobalt transport has been demonstrated experimentally. *hupE* mutants of *Synechocystis* PCC 6803 could only be rescued by the addition of cobalt or methionine to the cultures [73]. HupE/UreJ proteins are thought to contain 6 transmembrane helices with a signature motif sequence (HPXXGXDH) in the first transmembrane helix [74].

##### 5.1.1.6 CorA as a cobalt transporter

CorA is a family of divalent cation transporters that primarily transport magnesium, with nickel and cobalt as secondary metals, the binding affinities of which are 15–20 μM, 200–400 μM and 20–40 μM respectively [75,76]. As these concentrations of nickel and cobalt are not typically present physiologically, CorA is primarily a magnesium transporter. However, one CorA transporter from *Thermotoga maritima* has been found to select for cobalt over magnesium when present at 100-fold lower concentrations [77]. CorA itself is a homopentameric protein with each subunit formed of 8 helices that come together to form a funnel shape [78]. In organisms such as *E. coli* which have no dedicated cobalt transport system, CorA is likely to be the main source of cobalt entry into the cytoplasm.

#### 5.1.2 Cobalt efflux

Whereas cobalt import systems are regulated by a cobalt-dependent product such as adenosylcobalamin, cobalt efflux systems are generally regulated by the cobalt ion itself. Cobalt efflux proteins can be divided into two groups: the major facilitator superfamily (MFS) permeases and resistance nodulation division (RND) pumps (Table 1).

##### 5.1.2.1 MFS permeases

As described in Section 5.2.1, RcnR regulates the expression of RcnA, a Co(II)/Ni(II) efflux pump, that is responsible for detoxification of cobalt and nickel. Other members of this group are P_1B-4_-ATPases, a family for transmembrane metal transporters involved in transition metal homeostasis. Such transporters include CoaT as well as CtpD and CtpJ from *Mycobacterium smegmatis* and *Mycobacterium tuberculosis* respectively. These proteins have been demonstrated to be involved in cobalt homeostasis through cobalt export [79,80]. Both proteins contain six transmembrane domains with signature motifs SPC and HEG(S/G)T found in TM4 and TM6 respectively.

PmtA has been found to function as a Fe(II) and Co(II) efflux pump in *Streptococcus suis* whose expression is regulated by Fe(II), Co(II) and Ni(II) [81]. Deletion of *pmtA* resulted in increased Fe(II) and Co(II) sensitivity and accumulation of these metals. PmtA bears similarity to other P_1B-4_-ATPases such as PfeT from *B. subtilis* that is responsible for Fe(II) and Co(II) efflux [82].

##### 5.1.2.2 RND pumps

*Cuprivadius metallidurans* contains two RND pumps to mediate cobalt efflux: the cobalt, zinc and cadmium resistance system (CzcABC) and the cobalt-nickel resistance system (CnrABC): their mechanisms of regulation are described in 5.2.4, 5.2.5, respectively.

### 5.2 Protein-based cobalt sensors

**Fig. 5:**
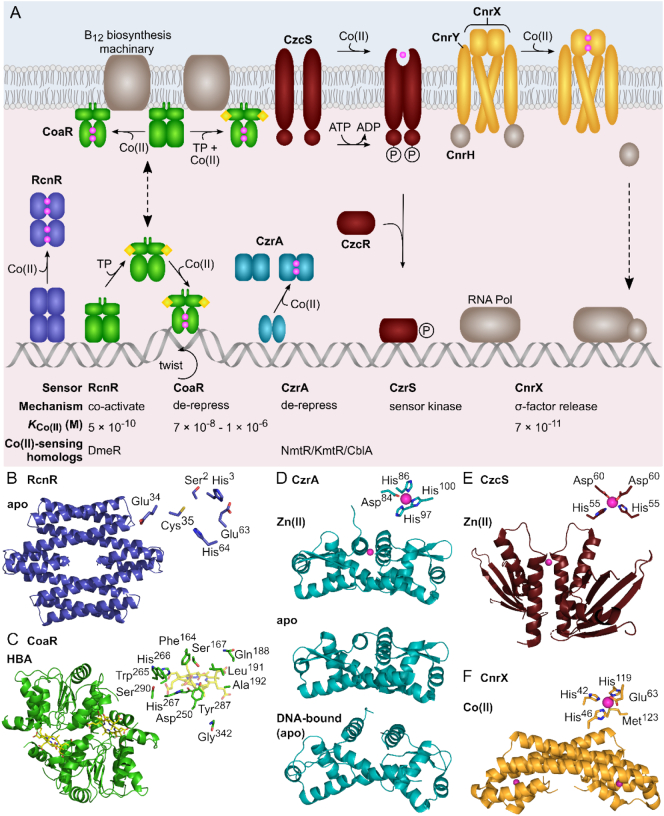
Protein-based cobalt sensors. A, RcnR represses expression of *rcnRAB* (in *E. coli* and Salmonella, two RcnR tetramers bind the target site). RcnR can bind four Co(II) ions per tetramer, which weakens the affinity for DNA, de-repressing expression [83]. CoaR binds the *coaT* promoter in the absence of effector. Co(II) binding is predicted to induce a conformational change which distorts the operator-promoter, enabling recruitment of RNA polymerase and activation of expression. CoaR also harbours a tetrapyrrole (TP) binding domain including a hydrophobic patch which is capable of interacting with membranes [96]. Tetrapyrrole binding might tighten the affinity of CoaR for Co(II), and/or association with membrane-associated B_12_ biosynthetic machinery could confer a kinetic advantage for Co(II) and/or tetrapyrrole acquisition. CzrA represses expression of *czrAB*, and Co(II) binding weakens the DNA affinity of the CzrA dimer, alleviating repression. The structures of apo- and Zn(II)-CzrA are similar in the absence of DNA and an entropic mechanism for allosteric regulation has been demonstrated [103]. CzcS sensor histidine kinase detects periplasmic Co(II), and metal-binding induces autophosphorylation at a conserved histidine by Mg(II)-GTP. Transphosphorylation of the response regulator CzcR induces a conformational change promoting DNA-binding and activation of *czcCBA* [113]. CnrX is a transmembrane protein which forms a complex with CnrY and cytoplasmic CnrH. Co(II) binding to the periplasmic domain of CnrX, induces a conformational change which is transduced through the complex, releasing the CnrH extracytoplasmic function sigma factor. CnrH is recruited by RNA polymerase enabling expression of cnrCBA [112,113]. B–F, structural models of Co(II)-sensing proteins. B, Structural representation of RcnR tetramer (dimer of dimers) generated from PDB 5LYC. The ligands identified for co-ordination of Co(II) are shown. C, Dimeric representation of the deduced tetrapyrrole binding domain of CoaR bound to hydrogenobyrinic acid (HBA), modelled on the structure of CobH precorrin isomerase (PDB 1I1H) as described in [96] (main chain of Gly^342^ shown). The CoaR ligands which spatially overlay with the HBA-binding site of CobH are shown. D, The structures of Zn(II)- (PDB 2M30) and apo-CzrA (PDB 1R1U) reveal similar ‘open’ conformations which contrasts with the ‘closed’ conformation of the DNA-bound form (PDB 2KJB) [103]. The Zn(II) (and Co(II)) binding site at the dimer interface is shown. E, The sensor domain of *P. aeruginosa* Zn(II)-CzcS (PDB 5GPO) suggests Zn(II) binds to reciprocal residues at the dimer interface. His^55^, but not Asp^60^, is conserved in the Co(II)-responsive CzcS homolog from C. metallidurans [114]. F, The soluble periplasmic domain of CnrX (PDB 2Y3B) displays a similar structural fold to MerR-family sensors such as RcnR. The Co(II)-binding site is shown: a sulphur donor ligand is provided by a conserved methionine in contrast to the invariant cysteine in RcnR [83,120].

Protein-based cobalt sensors are typically cytoplasmic proteins which both sense intracellular cobalt and regulate gene expression, although transmembrane proteins which can sense periplasmic cobalt have also been described (Fig. 5). These sensors modulate the expression of cobalt-efflux systems (or in one case a cobalt-requiring enzyme) as cobalt availability increases. No protein-based cobalt sensor has, as yet, been identified that regulates expression of a cobalt importer, with import commonly regulated *via* negative feedback from a cobalt-containing biosynthetic product such as vitamin B_12_.

#### 5.2.1 CsoR/RcnR-family: RcnR/DmeR

The best characterised cytoplasmic Co(II)-sensor belongs to the CsoR/RcnR-family of metal-dependent de-repressors [83]. These sensors typically repress the expression of transporters required for metal-export from the cell. DNA binding is inhibited upon metal co-ordination, relieving repression and enabling the removal of excess metal. RcnR was initially identified in *E. coli* as a regulator of the *rcnR-rcnAB* divergon, encoding the Co(II)/Ni(II)-efflux pump, RcnA (Section 5.1.2.1), and periplasmic protein, RcnB, plus autoregulation of its own expression [84]. RcnR is a tetrameric α-helical dimer of dimers that responds to both Co(II) and Ni(II) in the cell (Fig. 5b) [85]. Binding of the cognate metals is *via* a six-coordinate site and requires a cysteine (invariant in the CsoR/RcnR-family) and 5 O/N donor ligands (Fig. 5b) [86]. Co(II)-binding recruits, and orders, the amino terminus enabling a cross-link between subunits to drive allostery [86,87].

RcnR, and the homologous protein, DmeR, have also been functionally characterised in organisms which can synthesise B_12_ *de novo* such as the human pathogen *Salmonella*, the leguminous plant symbionts *Rhizobium leguminosarum and Sinorhizobium meliloti*, and plant pathogen *Agrobacterium tumefaciens* [[88], [89], [90], [91], [92]]. In each case the genetic architecture differs from *E. coli* (which does not synthesise B_12_)*:* in *Salmonella*, *rcnB* is not part of the *rcnR* operon, and in the plant microbes, DmeR regulates expression of *dmeF*, encoding a cation diffusion facilitator Ni(II)/Co(II)-efflux pump, distinct from RcnA [88,89,91]. The binding affinity, or stability of the Co(II)-RcnR complex as a *K*_D_, for *Salmonella* RcnR is 5 × 10^−10^ M. Autoregulation by *Salmonella* RcnR confers hysteresis: This dampens the response to elevated cobalt and shifts the dynamic range away from what might be predicted from metal-affinity alone [93]. How Co(II)-sensing by RcnR/DmeR relates to B_12_-production in these organisms remains to be investigated.

#### 5.2.2 MerR-family: CoaR

CoaR is a Co(II)-sensing MerR-family regulator from the cyanobacterium *Synechocystis* PCC 6803 [94] (Fig. 5a). Regulation by MerR-family sensors is characterised by sub-optimal spacing of the −10 and −35 promoter elements upstream of the target gene. These sensors bind their target DNA sequence with only a modest change in affinity between the apo- and holo-forms, and metal-binding causes a conformational change which under-winds the DNA allowing recruitment of RNA polymerase [95]. During exposure of *Synechocystis* to increased Co(II) (but not other metals), CoaR activates expression of *coaT* encoding a Co(II)-effluxing P_1_-type ATPase (Section 5.1.2.1) [94]. A Cys-His-Cys motif at the carboxy terminus of CoaR is implicated in Co(II)-coordination. CoaR also contains a putative tetrapyrrole-binding domain with homology to CbiC/CobH, the enzyme which catalyses the 1, 5-sigmatropic migration of a methyl group of precorrin-8 to generate hydrogenobyrinic acid during the aerobic biosynthesis of vitamin B_12_ (Fig. 5c) [96]. *Synechocystis* synthesises B_12_ *via* the anaerobic pathway and *cbiE* mutants, which will accumulate B_12_-precursors prior to cobalt-precorrin 7, demonstrate altered expression of *coaT* [94]. When expressed in *E. coli*, Co(II)-dependent activation of CoaR also requires residues predicted for tetrapyrrole binding, in addition to those which form the cobalt site [96]. One model is that CoaR has dual effectors in order to somehow coordinate both the levels of Co(II) and B_12_ (or precursor) in the regulation of Co(II)-export.

Thermodynamic characterisation of multiple metal sensors in *Synechocystis* reveals that Zn(II)-sensors ZiaR and Zur bind Co(II) at least 100-fold more tightly than does CoaR (CoaR *K*_Co(II)_ = 7 × 10^−8^–1 × 10^−6^ M), and Co(II) is able to activate the allosteric response of both Zn(II) sensors [96]. This raises the question: how is CoaR more competent than ZiaR and Zur at responding to Co(II) *in vivo*? Notably, metal-sensitivity is not solely a function of metal-affinity but also access, allostery, affinity for DNA, and abundance [57]. Sequence analysis of CoaR reveals a carboxy-terminal hydrophobic region predicted to interact with membranes [94,96]. Membrane association with CoaR could confer a kinetic advantage to Co(II)-sensing by CoaR over ZiaR and Zur in effect providing preferential access to cobalt [96]. Several components of the cobalamin biosynthetic pathway are thought to be membrane associated and could therefore encourage formation of a membrane-based metabolon [97,98], limiting the release of precursors during synthesis. The possibility that CoaR co-localises with this machinery, perhaps *via* its precorrin isomerase-like domain, and that this promotes channelling of Co(II) and/or a B_12_ precursor to CoaR, requires further investigation. In future, the magnitude of such kinetic effects could be quantified by reference to a thermodynamic framework such as that set out in Section 6.5.4. Progress in understanding the metalation of CobW (Section 6.6) highlights the merits of such an approach: By further analogy to CobW, the possibility that the Co(II) affinity of CoaR is enhanced by interaction of its precorrin isomerase domain with a tetrapyrrole or other partner, should also be considered.

#### 5.2.3 ArsR-SmtB family: NmtR/KmtR/CzrA/CblA

The ArsR-SmtB family of metal sensors are particularly widespread in bacteria and two examples which sense Co(II) (and Ni(II)) have been identified in *Mycobacterium tuberculosis*: NmtR and KmtR [99,100]. NmtR and KmtR repress expression of a Co(II)/Ni(II)-exporting P_1_-type ATPase and a cation diffuser facilitator metal-efflux pump, respectively. Upon metal co-ordination (which is distinct for each sensor [99,100]), the affinity for their respective DNA binding site is weakened, resulting in de-repression of the downstream gene [101]. Characterisation of NmtR and KmtR has revealed facets of metal-sensing that highlight the difficulties in designating the cognate metal(s) to metal sensor proteins and the vital contribution of metal-access, that is intracellular metal-bioavailability, to metal-specificity.

Repression by both NmtR and KmtR is alleviated by Co(II) and Ni(II) in mycobacterial cells. However, during heterologous expression in cyanobacteria, NmtR responds solely to Co(II) and not Ni(II). This suggests that the different cell types have distinct metal availabilities equating to differences in available free energies, see Section 6.6, and/or altered access due to metal channelling [99].

When mycobacterial cells are grown in minimal medium, both KmtR and NmtR are responsive to the cognate metals. However, when cultivated in a complex growth medium, expression of the gene regulated by KmtR is already elevated and unaffected by metals. In contrast, NmtR retains Co(II) and Ni(II)-responsiveness [99]. The prediction that KmtR is more sensitive than NmtR to Co(II) and Ni(II) was tested by competition between the two proteins *in vitro*: KmtR was able to out-compete NmtR for both Co(II) and Ni(II) consistent with a tighter metal affinity and increased sensitivity *in vivo* [99]. This could enable a graded stress response to increasing Co(II) (and Ni(II)) in mycobacteria reflecting discrete functions of the two transporters.

*Staphylococcus aureus* CzrA demonstrates selectivity towards Zn(II) and Co(II) *in vitro* and *in vivo* [100,102]. Allosteric regulation requires a tetrahedrally co-ordinated metal ion afforded by Co(II) and Zn(II), but not Ni(II) (Fig. 5a,d) [100]. The contribution of allostery to the selectivity of metal-sensor proteins (even within the same family) is highlighted by comparing this with the allosteric site of NmtR: here a six-coordinate site is required to trigger the allosteric mechanism, discriminating against Zn(II) (despite NmtR having a tight Zn(II) affinity) [100]. Structural determinations of apo- and holo-CzrA reveal similar “open” conformations in contrast to the “closed” conformation of the DNA-bound apo-structure [103]. Rather than a major structural reorganisation, cognate metal ion binding to CzrA is entropically driven and alters the internal protein dynamics [103]. A contribution from solvent entropy to allostery has also been established suggesting a model where dynamic redistribution between CzrA and surface water molecules occurs without significant perturbations in the protein structure [104]. This may be a general model for metal-sensing by members of the ArsR/SmtB family which undergo only small structural changes upon metal-binding.

Recently, a cobalt-sensing member of the ArsR-SmtB family has been characterised in *Rhodococcus rhodochrous* [105]. Atypically, rather than regulating metal-export machinery, CblA de-represses expression of a cobalt-dependant nitrile hydratase (NHase) in response to cobalt and nickel [106]. NHases are of interest to industrial biotechnology for the large-scale production of acrylamide and nicotinamide in addition to their relevance for plant-microbial interactions [107]. This is the first description of a NHase being regulated by cobalt in addition to substrate-dependant activation of transcription. Cobalt NHases have a non-corrin catalytic Co(III) at their active site [108]. A role for trapping kinetically stable Co(III) ions in the detection of cobalt by CblA is an intriguing possibility.

#### 5.2.4 Two-component regulatory system: CzcRS

The plasmid-borne resistance to *cadmium-zinc-cobalt* (*czc*) determinant originally identified in *Cupriavidus metallidurans* encodes genes for the CzcABC resistance nodulation and cell division (RND) exporter (Section 5.1.2.2) [109]. Expression is induced by Co(II), Zn(II), and Cd(II) *in vivo* [110,111]. The regulatory mechanism includes CzcR (response regulator) and CzcS (sensor histidine kinase) as a two-component regulatory system [112,113] (Fig. 5a). In contrast to cytoplasmic metal sensors, metal detection is by the periplasmic sensing domain of CzcS which initiates a signalling cascade, and phosphorylation of CzcR. Activation of CzcR leads to transcriptional activation of *czcABC*. The structure of the Zn(II)-bound CzcS sensor domain from *Pseudomonas aeruginosa* has been determined revealing that a His_2_Asp_2_ tetrahedral site could form at the dimer interface (Fig. 5e). However, CzcS from *P. aeruginosa* appears to be distinct from that of *C. metallidurans*, as the former does not respond to Co(II) *in vivo* [114], despite CzcA providing resistance to Co(II) in both organisms. Mutation of the metal-binding site in *P. aeruginosa* CzcS reduces Zn(II) sensing and substitution of the Asp ligands with Cys enables Co(II) detection *in vivo* [109,115]. Sequence differences between CzcS protein, and/or different metal availabilities in the periplasm, may explain the distinct metal-specificities of CzcS sensors in each organism.

#### 5.2.5 Transmembrane anti-sigma factor: CnrYXH

In addition to CzcS, *C. metallidurans* senses periplasmic Co(II) (and Ni(II)) by the transmembrane signal transduction system CnrYXH (Fig. 5a) [116]. CnrX is a transmembrane protein with a metal-sensing domain that extends into the periplasm [117]. CnrX interacts with the transmembrane protein CnrY, an anti-sigma factor which sequesters the extra-cytoplasmatic function sigma factor CnrH at the membrane [118,119]. Binding of Co(II) or Ni(II) to CnrX induces a conformational change which is transduced to CnrY, weakening the interaction with CnrH. Cytoplasmic release of CnrH allows recruitment by RNA polymerase, promoter recognition and expression of *cnrABC* [119]. CnrABC, a RND efflux pump confers Co(II) and Ni(II) resistance to *C. metallidurans* (Section 5.1.2.2) [119]. The structure of the holo-CnrX soluble periplasmic domain reveals a dimer with an octahedrally co-ordinated Co(II) or Ni(II) ion [117,120]. Signal transduction by CnrX requires a conserved methionine (Met^123^) which is also recruited (*via* the thioether sulphur) to the metal-binding site by Co(II) and Ni(II) (Fig. 5f) [117,120]. Zn(II) adopts a trigonal bipyramidal binding site not involving Met^123^ and Zn(II)-CnrX is non-functional [117]. Intriguingly, the helical bundle of CnrX displays structural similarity to RcnR/CsoR-family cytoplasmic metal sensors (Fig. 5a, f). These sensors use an invariant cysteine as a metal ligand [83]. Met^123^ is conserved among CnrX homologs and although an uncommon metal ligand, is considered to be more suited to the redox environment of the periplasm. Methionine has been observed in the periplasmic metal-binding site of Cu(I)-exporting RND pump CusABC, divalent metal transporter NRAMP and periplasmic chaperone CopC [[121], [122], [123]]. Substitution of Met^123^ with cysteine in CnrX abolishes Ni(II)-responsive expression and decreases the Co(II)-affinity by ~2000-fold [120]. Spectroscopic analysis suggests Cys^123^ is not involved in Co(II) co-ordination. Interestingly, the Zn(II) affinity of wild type CnrX is ~3-fold weaker than the affinity for Co(II) and this remains unchanged in the Met to Cys substitution [120]. The use of methionine in metal-coordination may contribute to allosteric metal-selectivity [117,120].

While the metal affinities of many cytosolic metal sensors have been determined, few are known for extra-cytoplasmic metal sensors likely due to the analytical challenge of working with membrane-bound proteins. For cytoplasmic sensors, these metal-affinities can be used to infer intracellular metal availabilities (Section 6.5), but it is less clear whether the same relationship exists for extra-cytoplasmic metal sensors and metal availabilities. One option for estimating Co(II) availability in the extra-cytoplasmic space is to measure transport rates of Co(II)-importers (Section 5.1.1). Co(II)-uptake assays with CbiMNQO and NhlF suggest *K*_m_ values in the range of 10^−7^ to 10^−8^ M [124]. In Gram-negative bacteria, controlling metal availability in the periplasm presents a greater challenge than for the cytoplasm: the periplasm is more vulnerable to external fluctuations than the cytosol and metal ions may diffuse through outer membrane porins [125]. The outer membrane does afford some protection against the extracellular environment but there may be regions which are contiguous with the extracellular environment. Despite this, the Co(II) and Ni(II) affinities of the CnrX soluble periplasmic domain have been determined to be 6.5 × 10^−11^ M and 1.7 × 10^−12^ M, respectively [120,126]. These affinities are notably tight and suggest that the availability of metal ions in the periplasm may be somewhat analogous to the cytoplasm (Section 6.5). Metal speciation in the extra-cytoplasmic space is not well defined although there is a suggestion that copper could be more available than in the cytosol (Section 6.3), perhaps relating to a predominance of cupric rather than cuprous ions. Low molecular weight molecules (*e.g.* amino acids, glutathione, citrate), and adventitious sites on proteins and membrane components are candidates for chelating and buffering metals in this compartment. Glutathione is exported into the periplasm of Gram-negative bacteria, and can buffer Cu(I) below 10^−15^ M when in excess [127,128]. The substrate for NikA-mediated Ni(II)-import in *E. coli* and *Helicobacter pylori* is a Ni(II)-histidine_2_ complex which might suggest the presence of histidine in a bacterial periplasm [129]. Histidine is implicated in the speciation of Ni(II) and Zn(II) in labile metal pools [130,131]. By analogy to cytoplasmic metal sensors, knowledge of the metal availabilities to which sensors are tuned (Section 6.5), should advance understanding of how metals, including Co(II), are discerned by extra-cytoplasmic proteins.

#### 5.2.6 Metal sensors are fine-tuned to a narrow dynamic range

The Co(II)-sensor RcnR can bind Zn(II) *in vitro* and DNA-binding is impaired by Zn(II) [90]. By measuring affinities for both Co(II) and Zn(II), along with affinities for DNA of the apo- and metal-bound forms of the protein, sensitivities to both metals were calculated [90] (also see Section 6.5.1). Counterintuitively, RcnR is about two-orders of magnitude more sensitive to Zn(II) than to Co(II), yet *in vivo* RcnR does not respond to non-inhibitory levels of Zn(II). A sensor that does respond to Zn(II) *in vivo*, Zur, is reciprocally competent to respond to Co(II) *in vitro* but fails to do so under normal conditions in living cells. Importantly, the *bona fide* sensors are only about an order of magnitude more sensitive to the cognate metal compared to the non-cognate sensors [90]. Thus, the sensors have evolved to show perfect discrimination only within a narrow range of metal concentrations.

Variants of the Ni(II) sensor InrS with weakened Ni(II) affinities retained the ability to dissociate from DNA *in vitro* but lost the ability to detect sub-inhibitory elevated Ni(II) *in vivo* [132]. Again, only a modest (about ten-fold) change in sensitivity was enough to lose detection of the cognate metal. Subsequent experiments showed that the dynamic range of InrS was poised to track with the degree of Ni(II)-saturation of a cytosolic-like buffer (Section 6.5.2). Under such a regime, modest changes in metal concentration can reflect large changes in the total number of atoms bound within the buffer. In this manner, sensors can be set to detect when the buffer approaches metal-saturation or -depletion to trigger, for example, export or uptake respectively.

### 5.3 Riboswitches for B_12_ and for cobalt

Riboswitches are allosteric RNA molecules, present in the upstream region of bacterial mRNAs, and whose conformation is modified upon selective binding of a specific inducer. Riboswitches are generally split into two domains: an aptamer which recognises the effector molecule and an expression platform which modulates the expression of the downstream gene(s). Effector binding to the aptamer domain induces a conformational change in the expression platform leading to transcription termination, translation initiation, message stability, or alternative splicing of the associated transcript [133]. Characterised riboswitches present a diverse repertoire of regulatory mechanisms and effectors: the first effector described being B_12_ and now more recently the identification of riboswitches for metal ions.

#### 5.3.1 The B_12_ riboswitch

To test the credibility of an “RNA World” experiments were performed to establish whether the catalytic repertoire of RNA enzymes could readily be extended beyond the limited hydrolytic activities known in nature, for example of self-splicing introns [134]. In 1990, the Systematic Evolution of Ligands by EXponential enrichment (SELEX) approach was established which identified, from a random pool of oligonucleotides, single stranded nucleic acids that could fold into structures (aptamers) with high affinity and specificity for small biological molecules [135,136]. Despite the presence of only four nucleotides, single-stranded nucleic acid aptamers have the versatility to adopt structures with comparable ligand binding affinities and specificities to proteins. A B_12_-binding aptamer was identified from a pool of 10^15^ molecules in 1994 and bound cyanocobalamin with tight affinity (*K*_D_ ~ 9 × 10^−8^ M) but with negligible affinity for adenosylcobalamin (AdoCbl), highlighting the degree of specificity that can be afforded by an RNA aptamer [137]. These data supported the notion that B_12_ could have been an early evolutionary cofactor with a role during the transition from the RNA-dominated biological world to the present, where DNA and proteins prevail [26].

The verification that RNA molecules could bind biological targets led to the pursuit of naturally occurring RNAs as regulatory elements, now known as riboswitches. The first riboswitch was identified in the 5′-untranslated region of *btuB* mRNA, encoding the outer membrane TonB-dependent B_12_-importer in *E. coli* and other bacteria [138]. Expression of *btuB* is down-regulated in the presence of B_12_ by inhibiting association of the ribosome with *btuB* mRNA [139]. No protein-based regulatory sensor which recognised B_12_ could be identified and the unusually long 5′-leader sequence, which forms secondary structure elements, is now known to be responsible for the allosteric regulation of *btuB* translation by B_12_ [138]. The *btuB* riboswitch is formed of a ~ 200 nucleotide B_12_-binding aptamer (receptor domain) and a ~ 40 nucleotide expression platform (regulatory domain) [140].

**Fig. 6:**
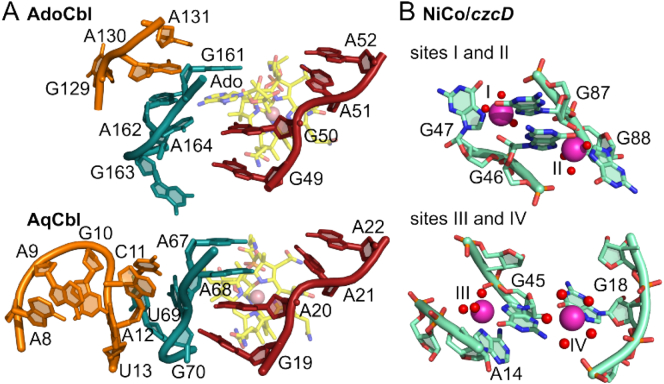
Riboswitches for B_12_ and Co(II). A, The binding site of AdoCbl and AqCbl in the AdoCbl riboswitch (Thermoanaerobacter tengcongensis; PDB 4GMA) and env8AqCbl riboswitch (marine metagenome; PDB 4FRN), respectively (adapted from [236]) . Although similar orientations of the J3/4 strand (red) are observed, the differential positioning of the central J6/3 strand (teal) enables accommodation of the deoxyadenosyl moiety (Ado) of AdoCbl which base pairs with A162 in the AdoCbl riboswitch, but is occluded by A20 and A68 in env8AqCbl [148]. B, The Co(II)-binding sites in the NiCo riboswitch from Erysipelotrichaceae bacterium (PDB 4RUM). The four Co(II) ions bind with octahedral co-ordination geometry and Co(II) ions I and II are co-ordinated by four conserved guanine nucleotides (G46, G47, G87, G88). Site III requires G45 which also forms the outer co-ordination sphere of site II implicating an allosteric linkage between sites I-III [148].

The complex secondary structure of the B_12_ riboswitch involves twelve helical domains and formation of a tertiary interaction with a kissing loop close to the B_12_ binding site [141]. Substrate binding by the receptor domain relies on shape complementarity, primarily through van der Waals contacts, with relatively few hydrogen bond interactions, and the kissing-loop is required for the allosteric conformational change in the regulatory domain to alter gene-expression [141,142]. The ligand binding pocket is formed by a four-way junction and specific nucleotide interactions to the 5′-deoxyadenosyl moiety of AdoCbl (Fig. 6a) [142]. Riboswitches with selectivity for different B_12_ derivatives have peripheral extensions which discriminate against AdoCbl by occluding the bulky adenosyl group in favour of smaller upper ligands as in aquacobalamin (AqCbl) and methylcobalamin (MeCbl) (Fig. 6b) [142]. It has recently been shown that Mg(II) can pre-organise the *btuB* riboswitch into a binding-competent conformation that recognises AdoCbl [143]. Binding affinities for B_12_ riboswitches range from 2.5 × 10^−7^ M for AdoCbl (*btuB* riboswitch) to 7.5 × 10^−9^ M for MeCbl (*env8AqCbl* riboswitch) [142]. However, the degree to which co-transcriptional folding might affect the affinity and selectivity of these riboswitches remains unknown.

When B_12_ levels are low, the *btuB* ribosome binding site (RBS) is available for ribosome binding, and translation proceeds. In the presence of AdoCbl, the ribosome binding site (RBS) is sequestered by an anti-RBS complementary sequence forming an intrinsic terminator which precludes translation initiation [142]. Selective interaction of the anti-RBS with the RBS or an anti-anti-RBS sequence, when bound or unbound by AdoCbl, respectively, enables the conformational switch necessary for B_12_-responsive translation of *btuB* [133]. Folding of the riboswitch into these alternate structures is aided by transcriptional pausing by RNA polymerase [144].

The B_12_ riboswitch is widespread in both Gram-negative and Gram-positive bacteria, associated with genes that are involved in B_12_-biosynthesis, −uptake, −utilisation and also cobalt-supply [21]. The sequence and secondary structure of the B_12_ aptamer domain is evolutionarily conserved. Genomic analysis using this sequence has identified genes encoding high-affinity uptake systems for Co(II) (*versus* for Ni(II) which have a NikR binding site) that are required for cobalt supply to B_12_ biosynthesis (Section 5.1.1), and B_12_-independent isoenzymes that can be utilised by prokaryotes when B_12_ is scarce [21,22]. In *Listeria monocytogenes* hierarchal regulation mediated by the B_12_ riboswitch has been observed [145]. The negative feedback regulation of B_12_ biosynthesis, mediated by the B_12_ riboswitch, maintains appropriate B_12_ levels in the cell. However, this might also limit the commercial production of B_12_ industrially using organisms for which limited genetic tools exist [146]. The possibility of engineering B_12_ biosynthetic pathways in heterologous, genetically tractable hosts where regulation can be easily modified (such as *E. coli*), present attractive alternatives.

#### 5.3.2 Riboswitches for cobalt

In addition to transcriptional regulating proteins (Section 5.2), cis-acting metal-responsive RNA molecules, “metalloriboswitches”, have also been proposed to selectively alter gene expression in response to Co(II), Ni(II), Mg(II), Mn(II) and Fe(II) [147]. These metalloriboswitches are typically located in the 5′-untranslated region of transcripts encoding metal transporters and in some cases act in concert with the protein-based metal sensors.

The *czcD* (NiCo) riboswitch was identified upstream of *czcD*, encoding a CDF-family transporter forming part of the cadmium-zinc-cobalt resistance determinant [148]. This riboswitch promotes expression of *czcD* in the presence of Co(II) and Ni(II), but not Mn(II) or Zn(II) in *Clostridium* species, suggesting a selective role in metal resistance *in vivo* [148]. Metal sensor proteins which selectively respond to Co(II) in the cell bind Zn(II) and Cu(I) more tightly than the cognate metal (in accordance with the Irving Williams series, (Section 6.2)), and in many cases, these noncognate metals also trigger allostery (for example RcnR and CoaR) [90,96]. Selectivity *in vivo* is enabled because the protein's affinity for Co(II) matches (is tuned to) the cellular availability of Co(II), but the affinities for Zn(II) and Cu(I) (although tighter than the affinity for Co(II)) are too weak to compete with the availability of these metals in the cell (Section 6.5). In contrast, affinities of the *czcD* riboswitch from *Clostridium botulinum* (*Cbo*) indicate that it binds Ni(II) and Co(II) more tightly than other metals (*K*_D_ values = 6.5 μM and 13 μM, respectively), with Mn(II) also binding weakly (*K*_*D*_ = 220 μM) with no binding of iron, copper or zinc detected [148]. Affinities were determined aerobically using in-line probing which monitors ligand induced structural changes in the RNA molecule, therefore values might represent a combination of affinity and allostery. It is possible that a metal ion can bind tightly but not induce the allosteric conformational change, as has been observed for some metal sensor proteins [149]. Furthermore, aerobic conditions are problematic when working with some metal ions, for example those which are sensitive to oxidation, such as Cu(I) and Fe(II). Compared to metal sensor proteins, the affinities of the *czcD* riboswitch for Co(II) and Ni(II) appear too weak to explain the selective response *in vivo* (Section 6.5).

The mechanism of regulation by the *czcD* riboswitch is *via* an intrinsic transcriptional terminator hairpin which prevents synthesis of the full-length mRNA in the absence of metal ions. Upon metal binding, the RNA is stabilised in a structure which promotes read-through of the full length transcript by RNA polymerase, and expression of *czcD* is enabled [148].

The structure of the *czcD* riboswitch from *Erysipelotrichaceae bacterium* (*Eba*) bound to Co(II) has been determined and reveals two pairs of coaxially stacked helices with no tertiary interactions [148]. Four Co(II) binding sites were identified (Fig. 6b). Two Co(II) ions (sites 1 and 2) are six-co-ordinate, each with three co-ordinating water molecules. Four highly conserved nucleotides (G46, G47, G87, G88) directly co-ordinate the two Co(II) ions. In each case co-ordination involves the N7 position (as well as 2′-OH for G46 and G87), the most nucleophilic RNA functional group which has also been implicated in metal ion co-ordination in other metalloriboswitches (Fig. 6b) [150]. Co-operative metal binding is observed for both *Eba-czcD* and *Cbo-czcD* riboswitches such that binding of the first Co(II) ion stabilises the second Co(II) site, with G46 and G47 providing a link in the primary co-ordination sphere of the two sites [148]. Two additional Co(II) sites are observed in the crystal structure, each co-coordinated by at least four water molecules: site three is linked to an outer co-ordination solvent molecule of site 2 *via* G45, with the fourth site possibly an artefact of the crystallisation conditions bound by only a single non-conserved nucleotide [148]. As such, the lability of the *czcD* riboswitch Co(II) sites is much greater than for metal sites of metal sensor proteins [151].

Recently, the *Eba-czcD* riboswitch has been biochemically characterised anaerobically using a fluorescent variant, and affinities determined under *in vitro* conditions where the metal ions have been buffered [151]. In this experimental setup, affinities of the *czcD* riboswitch were determined to be 120 nM for Co(II), 61 nM for Ni(II) and 11 μM for Mn(II). *In vitro* responses were also observed for Fe(II) and Zn(II) with affinities of 400 and 93 nM, respectively [151]. Here, there is closer agreement with the Irving Williams series than previously observed, and the results highlight the technical challenges of measuring metal affinities (Section 6.1). It is interesting to note that unlike metal sensor proteins, the metal binding sites formed by nucleic acids lack a sulphur donor, likely influencing metal binding affinities. When expressed in *E. coli*, the *Eba-czcD* riboswitch is activated by manganese, iron, cobalt, nickel and zinc, but when the *in vitro* affinities are compared with recent estimates of bacterial cellular metal availability, only Fe(II) and Mn(II) would be able to bind *czcD* in the cell [93,151]. Furthermore, the *czcD* riboswitch of *Listeria monocytogenes* appears to regulate a P_1B_-ATPase metal exporter (*LMO3448*) with homology to transporter PfeT, implicated in Fe(II)-efflux. Heterologous expression of *LMO3448* in *Bacillus subtilis* lacking *pfeT* provides resistance to Fe(II), and to some degree Mn(II) and Zn(II), but not to Co(II) or Zn(II) [151].

The cognate metal(s) detected by the *czcD* riboswitch remain unclear with conflicting data in different organisms and affinities for Co(II) being weaker than corresponding protein-based Co(II)-sensors (Section 5.2) [148,151]. That being said, sensors with relatively weak Co(II) affinities (~10^−7^ M) have been reported to respond to cobalt in the cell under certain conditions [92,96]. It is possible that the *czcD* riboswitch presents a mechanism of ‘last-resort’ which starts to respond as the cellular buffer for Co(II) (or other metal) becomes saturated. Conversely, it might respond to metal ions lower down the Irving Williams series where availability in the cell is in the nano- to micromolar range (Section 6.5). Robust characterisation of the *czcD* riboswitch in different organisms, as has been done for metal sensor proteins, is necessary to fully understand the role of the *czcD* riboswitch and which metal(s) it senses. This requires analyses of metal responsiveness *in vivo* under conditions which do not overwhelm cellular metal homeostasis, and adapting *in vitro* experimental designs which have been developed to biochemically characterise metalloproteins. It is possible that in different organisms, the *czcD* riboswitch responds to different metals depending on the metal availability and/or specific nucleotide differences which alter metal selectivity. In this way, the same RNA scaffold could have a different specificity for metal ions afforded by relatively small sequence changes (analogous to the selectivity of the B_12_ riboswitches for different B_12_ derivatives, and metal sensor proteins of the same family which detect different metals). Is it possible that metal-sites in RNA molecules are more rigid than those created by the side-chains of amino-acids, offering greater steric selection and hence departing from the constraints of the Irving-Williams series? As a relatively new area in the cell biology of metals, metalloriboswitches present novel questions.

### 5.4 Chaperones and chelatases for cobalt and B_12_

The selective advantages of metal delivery proteins *versus* metal insertases, metallochaperones *versus* metal-chelatases, overlap and distinctions blur. Viewed simplistically, chelatases distort their tetrapyrrole substrates to catalyse the insertion of cognate metals [152], while metallochaperones supply cognate metals to the active sites of proteins and cofactors and are necessary *in vivo* because of limited metal availabilities inside cells [153,154]: But chelatases can also confer a metal supply advantage. Reciprocally, some metallochaperones modify their partners to aid metal insertion. Vitamin B_12_ biosynthesis pathways utilise two remarkably different chelatases, the class II (CbiX/CbiK) and class I (CobNST) enzymes, for early- and late-stage cobalt insertion, respectively (detailed descriptions in 5.4.1, 5.4.2). The class II enzyme CbiK catalyses cobalt insertion into sirohydrochlorin at *in vivo* cobalt availabilities [93] and thus carries out both metal supply and insertion functions. The class I enzyme CobNST catalyses metal insertion into hydrogenobyrinic acid *a,c*-diamide when high concentrations of cobalt are supplied *in vitro* [155] but an additional aerobic B_12_ synthesis enzyme, CobW (Section 5.4.2.1), is necessary for cobalt supply *in vivo*, at least when cobalt is not in surplus [156]*.*

#### 5.4.1 The early cobalt chelatases and related enzymes associated with metal insertion

In the early cobalt insertion, or anaerobic, pathway cobalt is inserted into a macrocycle called sirohydrochlorin (Fig. 3a), an intermediate that represents a branch-point with a number of other modified tetrapyrroles including siroheme, coenzyme F_430_ and heme [10]. Indeed, metal insertion at this stage directs the intermediate to the corresponding final product. Thus, cobalt insertion directs the intermediate towards cobalamin biogenesis, whereas nickel insertion directs the intermediate towards coenzyme F_430_, whilst insertion of iron dictates the molecule is destined for either siroheme or heme synthesis. Metal insertion in all these cases is mediated by separate class II chelatase enzymes [157], all of which share a common structure and are clearly homologous. For cobalt insertion three separate enzymes have been identified and are known as CbiK [[158], [159], [160]], CbiX^L^ [160,161] and CbiX^S^ [157,162]. These enzymes insert Co^2+^ into sirohydrochlorin to generate Co(II)-sirohydrochlorin but are structurally related to the iron-inserting enzyme SirB that converts sirohydrochlorin into siroheme [157,163,164], as well as to CbfA, which is the nickel chelatase that inserts Ni(II) into sirohydrochlorin for coenzyme F_430_ biosynthesis [165,166].

**Fig. 7:**
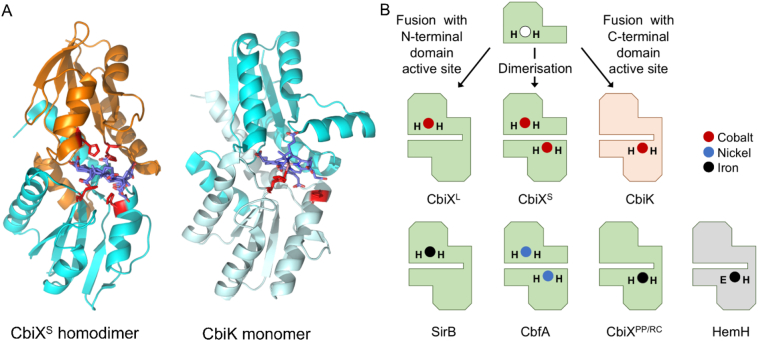
The chelatases associated with the cobalt-early pathway. (a) The type II chelatases are associated with the insertion of Co(II) into cobalamin, Fe(II) into siroheme and heme and Ni(II) into coenzyme F_430_. They all have a common fold despite often having limited sequence similarity. The CbiX^S^ cobaltochelatase from *Archaeoglobus fulgidus* is a homo-dimer composed of two identical subunits (coloured orange and cyan). The active site is formed at the junction of the two subunits and is symmetrical. Because of the symmetry imposed by having two identical subunits, sirohydrochlorin is sandwiched by two catalytic histidines from each subunit. In other type II chelatases, such as CbiK from *Salmonella*, the two subunits have fused together to give a single polypeptide chain and only two active site histidines (His 145 and 207) are found here – in this case located below the sirohydrochlorin substrate. The pseudo two-fold symmetry of CbiK, reflecting the ancestral CbiX^S^ heritage, is shown by the light and dark cyan colouring. (b) The evolution of the different type II chelatases is reflected in where the active site histidine residues are located. The cartoon highlights that the enzymes have the same basic architecture and are homologous, with those with a higher level of homology shown in green. For the small chelatases such as CbiX^S^ and CfbA the enzyme has a symmetrical active site with a total of 4 histidines, with two from each subunit. In the larger single subunit enzymes the two histidines are located either in the N-terminal domain of the protein or the C-terminal domain. Thus CbiX^L^ and CbiK (shown in pink) have evolved by retaining the active site histidine residues in different domains. Similarly, CbiX^PP/RC^ and SirB, which are both ferrochelatases associated with siroheme biogenesis, have also evolved with differential retention of the active site histidines. HemH (shown in grey), which is a ferrochelatase associated with heme biogenesis has changed one of the histidine residues for a glutamate and retained these in its C-terminal domain.

CbiX^S^ refers to a “small” CbiX enzyme, composed of around 120–145 amino acids and is generally found in the Archaea [162]. CbiX^S^ is active as a homo-dimer (Fig. 7a), with the active site formed at the junction between the two proteins. Consequently, the active site is symmetrical. Interestingly, the other class II chelatases CbiK and CbiX^L^ (the large form of CbiX) are about twice the size of CbiX^S^ and have N-terminal and C-terminal regions that separately align with the smaller protein. This indicates that both CbiK and CbiX^L^ have arisen following *cbiX*^*S*^ duplication [157]. Indeed, the structures of these larger enzymes confirm this finding, revealing a bilobal architecture composed of two α/β domains (Fig. 7a). The two lobes of the enzyme not only relate to one another by a pseudo-2-fold symmetry but also to the primordial CbiX^S^. The small CbiX is therefore considered as the “ancestral” class II chelatase [157].

The chelation reaction promoted by the sirohydrochlorin chelatases is mediated by a pair of conserved histidine residues. In the *Archaeoglobus fulgidus* CbiX^S^ enzyme these two conserved histidine residues are located at His10 and His74 [162]. In its dimeric form the CbiX^S^ complex therefore has a symmetrical active site that contains a total of four histidines. Two analogous histidines, His145 and His207 (Fig. 7a), are found only in the C-terminal domain of *Salmonella* CbiK and have been shown to be important for cobalt ion insertion indicating an asymmetrical active site [159,162,167]. In *Bacillus megaterium* CbiX^L^, the catalytically important histidine residues are found in the N-terminal domain of the protein (His14 and His79) indicating that this protein has evolved from a fusion of two CbiX^S^ in a different way to CbiK. Thus, the evolution of CbiK and CbiX^L^ can be followed by retention of the essential active site histidines within either the N- or C- terminal domain of these bilobal enzymes (Fig. 7b) [167]. Unexpectedly, CbiX^L^ was also found to contain an extended C-terminal extension that harbours both a 4Fe—4S centre as well as a long histidine-rich region. Deletion of this region does not affect the cobalt insertion reaction but it has been proposed that the region may be involved in metal ion storage and delivery [161]. However, it is clear that several types of chelatases contain Fe—S centres although it is not clear why this should be so as the centres are not required for catalytic activity [168,169]. Finally, another CbiX^L^-like protein from *Paracoccus pantotrophus* has been characterised and shown to be involved in siroheme synthesis – so technically this is a SirB. In this case the two catalytic histidine residues within CbiX^L-PP^ are found in the C-terminal region of the protein, which is also the closest ortholog to the *Rhodobacter capsulatus* CbiX that was shown to play a role in defending against photooxidative stress, possibly by altering flux through the tetrapyrrole pathway [170].

Duplication of the ancestral small chelatase CbiX^S^ has therefore led to the appearance in nature of a suite of enzymes including CbiX^L^, CbiK, CfbA and SirB that are able to mediate the insertion of divalent transition metal ions including Co(II), Fe(II) and Ni(II) (Fig. 7b). Indeed, most class II chelatases that insert metals into sirohydrochlorin appear to lack metal specificity and hence these enzymes will insert Fe(II), Ni(II) and Co(II) into the tetrapyrrole substrate [163]. It is not possible to predict which metal ion they are specific for from their tertiary structures and hence metal specificity is likely to be imposed within the cell depending on their binding affinity (see 6.2, 6.4, 6.5.4).

The type II chelatases are thought to function mechanistically by binding the tetrapyrrole substrate in a distorted fashion, thereby exposing the pyrrole nitrogens to allow them to deprotonate [152,167]. The deprotonation event may involve the active site histidine residues, which would then allow for direct metal insertion. However, it may be that the histidines are also associated with metal ion delivery to the active site to help promote catalysis through proximity. The distortion of the substrate coupled with the immediacy of the metal ion is all that is needed to promote the liganding event, which also occurs spontaneously at a slower rate in the absence of enzyme. Indeed, the insertion of various metal ions into sirohydrochlorin follows the Irving-Williams series in terms of relative rate, indicating that metal availability to the chelatase is a key determinant of metal selection [171].

The early insertion of cobalt into sirohydrochlorin generates cobalt-sirohydrochlorin (also known as cobalt-Factor II), and dictates that the molecule is destined for life as a cobamide. Cobalt-sirohydrochlorin next undergoes a series of methylation events that promote ring contraction prior to decarboxylation and methyl group migration and the amidation of the side chains, generating cobyric acid. The attachment of the lower nucleotide loop then produces the final cobamide [10].

#### 5.4.2 The late cobalt insertion pathway and the type I chelatases

The late cobalt insertion pathway proceeds from uroporphyrinogen III through the same series of methylation events as with the early insertion pathway, but obviously with cobalt-free intermediates (Fig. 3b) [10]. Cobalt is only added after the main corrin-ring has been generated, at an intermediate called hydrogenobyrinic acid *a,c*-diamide, which is the substrate for the late cobalt insertion chelatase (Fig. 3a) [172]. Unlike the monomeric ATP-independent chelatase of the early cobalt insertion pathway, this chelatase requires three proteins, CobN, CobS and CobT and consumes ATP. CobN is a large monomeric protein (120–150 kDa) that contains the main active site responsible for the transfer of the cobalt into the corrin substrate.

Hydrogenobyrinic acid *a,c*-diamide binds to CobN and the protein co-purifies with the substrate. CobS and CobT form a 450 kDa complex, which is required along with CobN to form a functional cobaltochelatase [172]. CobS and CobT share some similarity to each other and appear to form a two-tiered hexameric ring [173]. Whilst the CobS subunit contains a characteristic ATP binding Walker A and Walker B motifs belonging to the AAA+ superfamily of proteins, the CobT subunit has an integrin I domain, or von Willebrand factor, at its C-terminus, a motif that is usually associated with protein interactions associated with cell adhesion. CobS is thought to be the engine of the cobaltochelatase whereas CobT is more likely to be involved in the stabilisation of the motor complex platform [173].

The CobNST cobaltochelatase system is homologous to the magnesium chelatase system associated with the insertion of magnesium into protoporphyrin IX during the formation of bacteriochlorophyll (Bch) and chlorophyll (Chl) [174], which is catalysed by a very similar ATP-dependent heterotrimeric system consuming 15 ATP per round of catalysis [175]. The three proteins involved in the magnesium chelatase complex are named BchH/ChlH, a large monomeric subunit similar to CobN, BchD/ChlD that are the counterparts of CobT and finally BchI/ChlI subunits that belongs to the AAA+ (ATPases associated with diverse cellular activities) and are similar to CobS. This magnesium chelatase together with the cobalt chelatases form the Class I metal chelatase group [157]. In contrast to the sparse amount of information concerning the cobalt chelatase mechanism, many more studies have been undertaken on the magnesium chelatase.

The structure of *Synechocystis* PCC 6803 ChlH was solved in 2015 although no substrate complex was determined [176]. The protein is formed of six domains, including N-terminal ‘head’ and ‘neck’ regions and a central cage-like assembly. The ChlI subunits can self-assemble into a hexameric ring structure without ATP to resemble a 6-tooth rotor motor with an L-shaped helix to form the base and the outer ring for the motor [177]. The N-terminal domain of BchD/ChlD is similar to BchI/ChlI and its C-terminal domain contains a proline-rich region followed by a C-terminal integrin I domain [178]. This subunit also forms a hexameric ring but does not possess ATPase activity. As with CobS and CobT, the ChlD and ChlI form a double hexameric complex, which can be purified together and it was shown that the N-terminal domain of ChlD interacts with ChlI [179]. The 6-tooth rotor motor of BchI/ChlI is thought to twist upon ATP hydrolysis, mediating the movement *via* ChlD and its integrin I domain to bridge the ATPase activity through to the ChlH metal ion active site [180]. It is likely that similar conformational changes are also associated with the CobNST complex.

It is not fully understood why ATP hydrolysis is required for metal chelation within the class I chelatases. For cobalt insertion, the energy may be required to help promote insertion of the metal into the limited confines of a ring-contracted macrocycle. Moreover, the structure of the corrin substrate indicates that two of the opposite-facing pyrrole nitrogens remain protonated, thereby providing a steric block to the metal [15]. Hence, the cobalt chelation process into the corrin ring may require energy to distort the corrin macrocycle, promote proton abstraction and then encourage metal insertion. Unlike the type II chelatase, which can accept a range of metal substrates, CobNST is highly specific for cobalt and no other metals appear to function with the enzyme. Moreover, the type I cobaltochelatase requires a specific cobalt delivery system, a function performed by a protein called CobW (see Section 5.4.2.1).

##### 5.4.2.1 CobW and the COG0523 family of G3E GTPases

CobW was first identified as a component of the aerobic B_12_ pathway nearly three decades ago, when it was discovered that disruptions to the *cobW* gene impaired cobalamin biosynthesis in *Pseudomonas denitrificans* [181]. The functional role of CobW in Co(II) supply has finally been elucidated [156], with ambiguity about its metal-specificity resolved (Section 6.6).

CobW belongs to the COG0523 family, a subset of P-loop G3E GTPases whose common role is to support metallocentre assembly [182,183]. There are four branches of the G3E superfamily: MeaB, UreG, HypB and COG0523. MeaB, UreG and HypB have well-established functions: MeaB gates the insertion of B_12_ into methylmalonyl-CoA mutase (MCM) [184] while UreG and HypB support Ni(II)-acquisition by urease and hydrogenase, respectively [[185], [186], [187], [188]]. The genes encoding these proteins are generally found only once in a genome, in MCM-encoding, urease maturation and hydrogenase maturation gene clusters, respectively [183]. In contrast, diverse functions have been attributed to the COG0523 family which are found in numerous gene contexts and it is common to find more than one homolog within the same genome. This has led to the identification of at least 15 subgroups of the COG0523 family which differ in both function and metal-selectivity [183]. These include Nha3 which activates the Fe(III)-type nitrile hydratase (NHase) [189,190], numerous subgroups (including YeiR, YjiA, ZigA and ZagA) connected to Zn(II) homeostasis [130,[191], [192], [193], [194]], and CobW connected to vitamin B_12_ [181]. An estimated 12.5% of COG0523 genes are *cobW*, usually located adjacent to *cobN* in aerobic cobalamin biosynthesis gene clusters [183]. Given its homology to known metallochaperones, and genomic co-localisation with the cobaltochelatase gene *cobN*, a potential role as cobalt chaperone was suggested for CobW [195]. However, alternative functions (such as chaperone for the iron-requiring B_12_ enzyme CobG) were possible, particularly considering the roles of related COG0523 proteins in iron homeostasis. The mystery was solved when, by reference to intracellular metal-availabilities, CobW was shown to acquire Co(II) *in vivo* and its functional role in Co(II) supply was revealed [156] (Section 6.6).

COG0523 proteins are the largest of the G3E family. In addition to the highly conserved N-terminal domain which retains key structural features common to all GTPases [182,196], COG0523s contain a second, highly variable, C-terminal domain [183]. This is thought to interact specifically with client proteins with the high sequence variability suggesting a broad range of functional targets, as first indicated by gene context [183], and increasingly substantiated with experimental evidence [130,156,189,194]. The cobaltochelatase CobNST is the anticipated target for CobW, although no specific protein interaction has yet been demonstrated. Interestingly, while the Co(II)-acquisition site of CobW is likely localised to a conserved CxCC motif in the N-terminal domain [156] (Section 6.6), the C-terminal domain contains a conserved His/Asp-rich region [130] which could play a role in metal-handover.

## 6 A paradigm for metalation inside cells: CbiK and CobW

### 6.1 The determination of metal-protein affinities: A cautionary note

Widely used methods for determining the metal affinities of proteins can produce erroneous values [197]. Indeed, a substantial fraction of reported metal-protein affinities are incorrect [197,198]. As researchers follow published experimental precedents, so wrong values propagate and the ‘noise-to-signal’ ratio within the literature increasingly confounds the development and testing of hypotheses. Care will be needed to exclude unreliable values if metal affinities are interrogated by machine learning routines. Common issues include unrecognised upper or lower limits of experimental methods, inadvertent inclusion of competing ligands (*eg* buffers, salts), presence of metal-binding protein tags, poor pH control, the formation of adducts, non-equilibrium assays, oxidation of metal or ligand, contaminating metals, and misinterpretation of experimental data. To circumvent these pitfalls, robust methods for the determination of metal-protein affinities (usually *via* competition with well-characterised ligand standards) have been described [[197], [198], [199], [200]].

### 6.2 The metal affinities of CbiK do not match its requirement for Co(II)

The metal affinities of the CbiK cobalt chelatase of *Salmonella* were measured by competition against probes of known affinities: fura-2 for Co(II) and mag-fura-2 for Fe(II), Ni(II) and Zn(II), while the affinity for Mn(II) was too weak for either competitor [93]. An affinity for Cu(I) was obtained by competition against bicinchoninic acid. The affinity for Co(II) is similar to that for Zn(II) and slightly weaker than that for Ni(II), but Cu(I) forms substantially the most stable complexes. Viewed in isolation, these data indicate that CbiK might be vulnerable to mismetalation with other ions and perhaps preferentially Cu(I).

Because proteins are flexible, they offer negligible steric selection, at least in their nascent forms and in the absence of interacting partners (Section 6.5). Under these conditions the order of metal binding follows the Irving-Williams series [201]. Notably this was co-discovered by R.J.P. Williams who also proposed the entatic state (Section 1.1).

$$\mathrm{Mg}\left(\mathrm{II}\right)<\mathrm{Mn}\left(\mathrm{II}\right)<\mathrm{Fe}\left(\mathrm{II}\right)<\mathrm{Co}\left(\mathrm{II}\right)<\mathrm{Ni}\left(\mathrm{II}\right)<\mathrm{Cu}\left(\mathrm{II}\right)\;\left(\mathrm{Cu}\left(I\right)\right)>\mathrm{Zn}\left(\mathrm{II}\right)$$

The Irving–Williams series for divalent metals [201], plus Cu(I) and Mg(II). Note reversal of the less-than sign after copper. All first row metals can exist in the divalent form and Cu(I) ions, thought to predominate in the cytosol, also bind tightly, while Mg(II) binds weakly.

The order of metal binding to CbiK exemplifies the Irving-Williams series and highlights a universal challenge for metalation.

Mismetalation can involve a sub-set of ligands from the *bona fide* metal site, a different coordination geometry or even recruit additional ligands. However, the biological challenge is to populate each locus within metallo-proteins with the correct ions regardless of the coordination chemistries of aberrant binding. Inclusion of a metal within a cofactor such as a tetrapyrrole largely resolves these issues for the final metalloenzyme which can now contain a specific binding pocket that recognises the complex molecule. However, this still requires that the correct metal has previously partitioned into the tetrapyrrole (Section 6.4). A metal delivery protein or metallochaperone can resolve the specificity of metal insertion *via* specific protein-protein recognition between delivery protein and chelatase, but ultimately the challenge still remains: How does the correct metal, in this case Co(II), partition onto the delivery protein if other metals bind more tightly (6.4, 6.6)?

### 6.3 Bioavailability influences metal-protein speciation *in vivo*

A pair of proteins in the periplasm of a cyanobacterium share the same cupin fold, the same metal-binding ligands yet bind different metals from near opposing ends of the Irving-Williams series *in vivo*, manganese and copper [202]. Refolding of either apo-protein (MncA or CucA) *in vitro* in the presence of equimolar amounts of manganese and copper (either cuprous or cupric) led to binding of copper which inactivates oxalate oxidase activity of the manganese-protein [202]. A ten-thousand times excess of manganese is required at folding in order to correctly metalate the manganese-protein. This implies that manganese is at least ten thousand times more available than copper at the site where the nascent manganese-protein folds. Notably, while the cupro-protein is secreted in an unfolded state *via* the sec-machinery, the manganese-protein is secreted pre-folded *via* the Tat-machinery [[202], [203], [204]]. These data imply that the cytosol is a metal-protected environment where the bioavailability of the more competitive metals is controlled to prevent mismetalation of proteins that require less competitive ions.

The metal ions in MncA and CucA are buried with no, or negligible, access to solvent [202,205]. Within folded manganese-MncA metals become kinetically trapped. This implies that at some step along the folding pathway there is a nascent metal-binding site which requires a 10,000 times excess of manganese over copper to avoid entrapment of the incorrect ion. The MncA-related oxalate decarboxylase enzyme (OxdC) from *Bacillus subtilis* has been expressed in the cytosol of *E. coli* and found to entrap a range of inactivating metals [206]. For example, growth of cells in excess cobalt leads to the accumulation of cobaltous-MncA. The proteins of metallostasis must normally act to sustain the correct availabilities of the different elements to enable correct metalation of proteins and delivery pathways, including those that supply metals to tetrapyrroles and vitamin B_12_. But what are these vital availabilities: What defines intracellular metal-bioavailability and how can this parameter be measured?

### 6.4 CbiK as a paradigm for *in vivo* metalation

The cytosol is over-supplied with binding sites for essential metals [207,208]. This is also anticipated to be true in other biological compartments. Under these conditions proteins and other molecules compete for a limited supply of metals, rather than metals competing with each other for a limited supply of binding sites. To understand how Co(II) partitions onto CbiK, this competition was defined [93]. Reference values for cytosolic metal availabilities were generated (Section 6.5) against which the binding preferences of CbiK for different metals (Section 6.2) could be related.

### 6.5 Metal availability in the cell determined from the sensitivities of metal-sensors

It is straight-forward to determine the total amount of metal in a cell. By digesting a known number of cells in acid and then determining the concentration of each metal in the extract, for example by ICP-MS, the number of atoms per cell is readily calculated. Using the average volume of a cell, this can be converted into a pseudo concentration: Pseudo because many of these atoms will not be available in solution but sequestered in unavailable sites, kinetically trapped and/or compartmentalised. The crucial question is how much of each metal is accessible to nascent metalloproteins and how tightly must a protein (such as CbiK) bind in order to compete for each of these ions?

Metals such as Cu(I) and Zn(II) are at much lower availabilities than less-competitive (for binding to ligands) metals such as Co(II), Mn(II) and Fe(II) (Section 6.4) [93]. More than 20 years ago it was concluded that the cytosol contains essentially no ‘free’ Cu(I) or Zn(II) [207,209]. We take the term free to refer to the aquo-form (or any comparably weak ligands). To estimate the bioavailability of each metal a number of methodologies have been used including chemical probes, genetically encoded probes, and measurements of the metal affinities of cognate metal sensors [57,210].

**Fig. 8:**
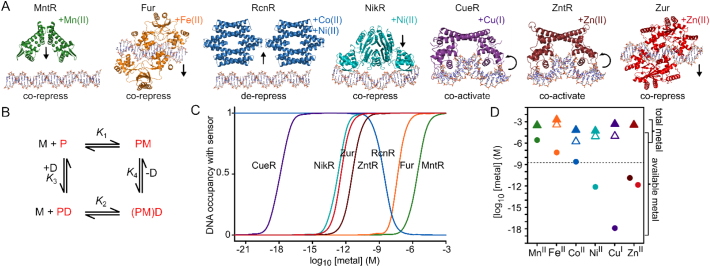
Thermodynamic characterisation of a set of sensors. A, The set of cytosolic protein-based metal sensors from Salmonella with cognate metal ion(s) and allosteric mechanism of regulation shown, as described in [93]. B, Thermodynamic cycle depicting the allosteric coupling of metal (M) binding and DNA (D) binding by metal sensor proteins (P) [211]. C, Fractional occupancy of target DNA binding site(s) with respective metal sensor protein as a function of buffered metal concentration, following determination of K_1__–__4_, and number of sensor molecules and target DNA-sites per cell (B), sensor protein abundance and number of target DNA binding sites as described in [93]. D, the ranges of total (triangles) and the available (buffered; circles) metal concentrations in Salmonella. Total metal was determined in cells grown in minimal media with (closed triangles) and without (open triangles) metal supplementation [93]. Open triangles obscured for Mn(II) and Zn(II). Available metal reflects the mid-point of each sensor's dynamic range in (c). The concentration equating to one hydrated atom per cell is shown by a dashed line.

DNA-binding metal-sensing transcriptional regulators have evolved to respond to the availability of a specific metal ion [90]. Metal co-ordination by the metal sensor induces a conformational change which allosterically activates or inhibits the interaction of the protein with its target DNA binding site to modulate downstream gene expression. Together, these systems enable the cell to mount an adaptive response to metals: achieving metal sufficiency whilst avoiding metal toxicity (5.1, 5). Each metal sensor is tuned to the cellular availability of its cognate metal and operates within a relatively narrow range (Section 5.2.6) [90]. If the sensitivity (which is determined by the allosteric mechanism and protein-abundance as well as metal-affinity, Section 6.5.1) of each metal sensor can be determined, it should become possible to determine the bioavailability of each metal in a cell (Section 6.5.3). To this end, a complete set of metal sensors was thermodynamically characterised in *Salmonella* (Fig. 8a) [93].

#### 6.5.1 Thermodynamic characterisation of a set of metal sensors

Metal binding to a metal sensor protein is thermodynamically coupled to DNA binding (Fig. 8b) [211]. As a consequence, the metal affinity of the DNA-bound form can be tighter or weaker than that of the sensor when free from DNA: in the case of the *Salmonella* Co(II)-sensor, the DNA affinity of Co(II)-RcnR is ~100-fold weaker than that of apo-RcnR [212], and this will be reciprocated by a ~100-fold weaker Co(II)-affinity of DNA-bound RcnR (*K*_2_ = (*K*_1_*K*_4_)/*K*_3_) (Fig. 8b). The opposite will be true for sensors whose DNA-affinity is tightened upon metal binding, for example co-repressors such as Zn(II)-sensing Zur. Ultimately, the sensitivity of a metal sensor depends on the population which associates with DNA (or activates the promoter in the case of co-activators) to alter gene expression [90]. Thus, the metal-affinity of the sensor when not bound to DNA (*K*_1_) is an imperfect approximation of sensitivity.

*Salmonella* metal sensors were identified for Mn(II), Fe(II), Co(II), Ni(II), Zn(II) and Cu(I) Fig. 8a [90,213,214]. For each sensor, the cognate metal-affinity (*K*_1_), and the DNA-affinities of the apo- and metalated-protein (*K*_3_ and *K*_4_) were measured *in vitro* (Fig. 8b) [93]. Notably, accurate metal-affinity measurements can be challenging (Section 6.1), due to the tight affinities exhibited by many metal-protein complexes (such as 10^−19^ M for Cu(I)-CueR) [93]. Informatic analyses and multiple reaction monitoring-mass spectrometry was used to determine the number of target DNA-binding sites and the number of sensor molecules per cell, respectively [93]. The thermodynamic cycle in Fig. 8b was solved predicting the DNA occupancy of each sensor as a function of buffered metal concentration [93]. This revealed that the buffered metal concentrations at which each sensor exhibited 0.5 of its response spanned twelve orders of magnitude from Mn(II) to Cu(I) (Fig. 8c) [93]. The cellular buffered metal concentration inferred from the mid-point of the dynamic range for each sensor is much lower than the total metal in a *Salmonella* cell when expressed as a concentration per cell volume. For Cu(I), Zn(II), Ni(II), and possibly Co(II), the buffered available metal concentration equates to less than one hydrated metal ion per cell (Fig. 8d) (Section 6.5.2). This is consistent with the hypothesis that the cell has an over-capacity to chelate metal [208].

#### 6.5.2 An associative cell biology of metals: Metal availabilities as free energy changes

The volume of an *E. coli* cell is about one femto litre. A single atom or molecule dissolved in this volume equates to a concentration of almost one nano molar (10^−8^ to 10^−9^ M). Estimates described in Section 6.5.1, and elsewhere [207,209,215,216], indicate that the available concentrations of many essential metals are sufficiently low as to equate to just a handful of atoms, or less than one hydrated atom, per cell (Fig. 8a). This has been a source of confusion: Perhaps metal availability must be considered at a population level with pico molar available Zn(II) equating to one atom in every thousandth cell; or perhaps the system must be time-resolved with one atom being available one thousandth of the time. Tremendous advances in technical abilities to observe molecular events in single live cells, make these explanations attractive. But the above arguments assume a dissociative cell biology of metals. An alternative hypothesis is that metals are widely delivered by specific protein-protein interactions *via* rapid ligand exchange reactions [57]. Again, progress in live cell imaging makes this explanation appealing. However, with such a large number of metalloproteins this implies an unfeasibly large number of undiscovered delivery systems. Moreover, this amplifies rather than solves the challenge associated with correct metalation since now the correct metals must somehow partition onto a multitude of delivery pathways.

**Fig. 9:**
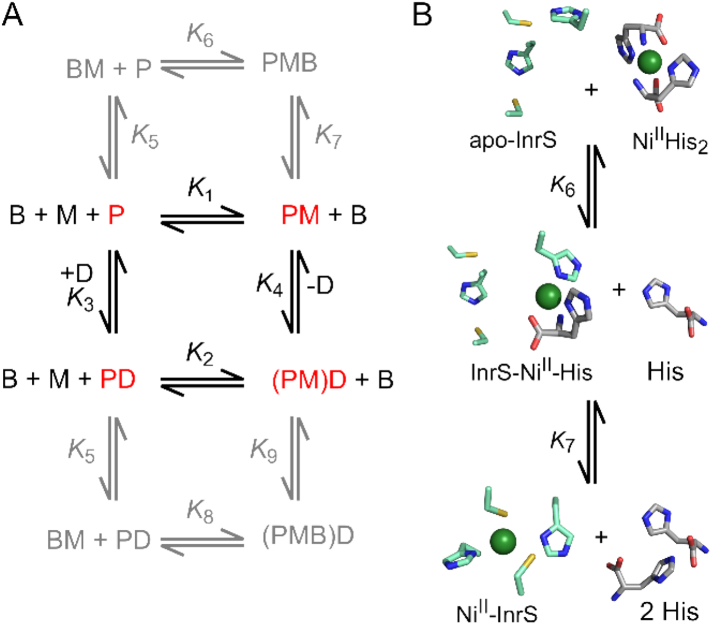
An associative cell biology of metals. A, The four allosteric end states of a metal sensor protein: apo-protein (P), metal-bound protein (PM), DNA-bound apo-protein (PD) and DNA-bound metal-protein ((PM)D). Metal (M) exchange occurs between the metal sensor protein and cellular buffer (B) *via* K_5_-K_9_ by an associative mechanism [57,93,211]. B, Proposed metal-acquisition by a metal-sensor protein (InrS, PDB 5FMN) from a cellular metal-buffer complex (Ni(*II*)His_2_, LHISNI01) *via* formation of a heterocomplex (modelled on PDB 4XKN). The Ni(II)-InrS model was generated using PDB 2HH7 plus free His (HIS_LFOH). InrS side chains are light blue, histidine from the cellular buffer is grey, nickel is green.

An associative cell biology of metals *via* buffered metal-pools provides an explanation (Fig. 9a) [57,93]. For example, a simplified cytosolic solution matching the intracellular concentrations of a dozen free amino acids and glutathione, was shown to buffer available Ni(II) such that the affinity of the Ni(II) sensor InrS was poised to track with metal saturation of the buffer, elaborated in Section 5.2.6 [132]. Histidine was the main competing species, and as noted in Section 5.2.5, some Ni(II)-importers such as NikABC, translocate Ni(II)-histidine complexes [129]. Fig. 9b shows atoms of Ni(II) in complex with histidine binding to InrS *via* associative ligand exchange. Such exchange reactions are rapid because they bypass the otherwise rate limiting step of release to the hydrated state. With such tight metal-protein affinities, off-rates to the hydrated state would be so slow as to exceed the lifetimes of proteins before thermodynamic equilibrium is approached: Associative ligand exchange overcomes this limitation.

It seems perverse to describe metal availabilities as the concentrations of almost non-existent hydrated species if the cell biology of metals is associative and the hydrated species irrelevant: But these values also provide information on the state of the relevant bound metal-species. Specifically, they indicate how tightly the metal is bound and hence which proteins can out-compete the buffer to acquire the metal by associative ligand-exchange. The terms chemical activity or chemical potential encompass this concept. To avoid confusion, cellular metal availabilities have been expressed as free energy changes, Δ*G*, associated with *in vivo* metal complex formation: the values for metal concentrations can be used to calculate the Δ*G* using the standard equation RTln*K*_A_, where *K*_A_ is the affinity of a molecule that would be 50% saturated with metal at the available buffered concentration [93]. These values provide a point of reference for understanding intracellular metalation.

#### 6.5.3 Intracellular metal availability follows the Irving-Williams series

**Fig. 10:**
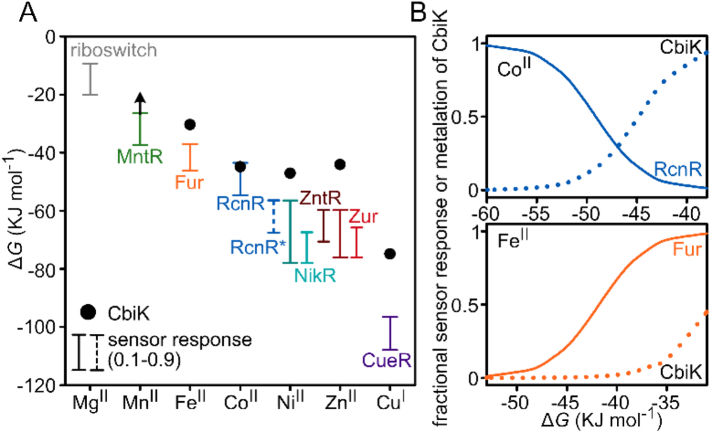
Metalation of CbiK. A, Metal availabilities (expressed as free-energy changes, ΔG) in the bacterial cytosol determined from the thermodynamic characterisation of a set of metal-sensor proteins and related riboswitch (Fig. 8) [93]. Bars show the ΔG range as each metal sensor shifts from 10 to 90% of its transcriptional response (*i.e.* 0.1–0.9 fractional DNA occupancy). In cases where there are two sensors for one metal, the combined range is also shown. An estimated Ni(II) availability from thermodynamic values for RcnR (RcnR*) is shown, using determined RcnR DNA-binding affinities and the Ni(II)-affinity of InrS, a related Ni(II)-sensor (as only a limit Ni(II) affinity is known for RcnR) [132,237]. The ΔG associated with metal binding to CbiK is also shown. The arrow for Mn(II) reflects a lower limiting value. B, Fractional occupancy of CbiK (dotted lines) with Co(II) (upper) or Fe(II) (lower) at the intracellular availabilities where RcnR and Fur (sold lines) shift from 1 to 99% of their responses, respectively.

Fig. 10a shows the intracellular metal availabilities (as Δ*G*) at the dynamic response ranges (0.1 to 0.9 fractional DNA occupancy) of the DNA-binding metal-sensors of *Salmonella* [93]. The sensors are tuned to the opposite of the Irving Williams series (Section 6.2) [93,201]: That is the most competitive metals are at the lowest Δ*G*, hence tuned to the lowest availabilities, while the less competitive ones are at less negative Δ*G* and hence tuned to the highest availabilities (Fig. 10a). As suggested in Section 6.3, intracellular metal availabilities compensate for the inherent metal-binding preferences of proteins. Under these conditions different proteins can acquire different metals determined by their ‘relative’ (with reference to the competing species within the intracellular milieu) metal-binding preferences, rather than absolute affinities.

The hypothesis that intracellular metal availabilities will be set to the opposite of the Irving-Williams series is not new [217]. A diversity of estimates of metal availabilities, based on the metal-affinities of proteins of metallostasis [207,209], or by using metal-responsive probes for example [218], are consistent with this notion. Moreover, the complex mixture of intracellular ligands (free amino acids, other small molecules, adventitious binding sites on the surface of macromolecules), that can be assembled into a diversity of coordination sites with limited geometric constraint, provides an ideal mixture which will inevitably buffer according to the Irving-Williams series [201]. It is anticipated that over the course of evolution metal-sensors and other proteins of metallostasis have been selected to limit saturation or depletion of this buffer. Similarly, metalloenzymes and proteins that acquire metals to supply cofactors will have evolved to obtain the correct metal in the context of these competing species.

#### 6.5.4 A thermodynamic framework for metalation predicts that CbiK acquires Co(II)

By calculating the Δ*G* associated with forming metal-CbiK complexes from the affinities described in Section 6.2, in comparison with the metal availabilities described in Section 6.5.3, it becomes evident that cobalt is poised to enable formation of Co(II)-CbiK (Fig. 10a). Other metals are estimated to bind CbiK more weakly than the competing intracellular species [93]. *In vitro* assays establish that sirohydrochlorin can spontaneously acquire cobalt *in vitro*. However non-catalysed insertion is negligible when Co(II) is buffered to an availability approximating the mid-point of the dynamic range for RcnR [93]. In contrast, under these buffered conditions insertion is sustained by CbiK, revealing the essential role of the chelatase for B_12_ biosynthesis in the context of competing intracellular species is noted in Section 6.4 [93]. By reference to this thermodynamic framework it becomes possible to understand why and how CbiK acquires the correct metal for the synthesis of vitamin B_12_.

Within the dynamic range of RcnR, Co(II) occupancy of CbiK is estimated to switch from entirely apo to fully saturated (Fig. 10b). This suggests that under some growth conditions Co(II) supply for vitamin B_12_ is limiting. Moreover, at the upper end of the dynamic range for Fur partial metalation of CbiK with iron becomes possible (Fig. 10a), albeit such mismetalation would be overcome by coincident supplementation with cobalt (Section 6.6). Notably, in mutants deficient in the chelatase for siroheme, CbiK can complement the deficiency and support iron-supply to siroheme [158]. Importantly, the data in Fig. 10a provides a thermodynamic framework within which it becomes possible to understand, and potentially optimise, the metalation of vitamin B_12_ and indeed other metal-dependent proteins and pathways.

### 6.6 Metalation of CobW

#### 6.6.1 Identification of the cognate metal

CobW contains a CxCC sequence motif which is highly conserved in the COG0523 family [183] (Section 5.4.2.1). Mutational analysis has confirmed that the CxCC sequence is necessary for metal-handling activities of various COG0523 proteins including *in vivo* activation of Fe(III)-type NHase by Nha3 [190], the protective effect of YeiR under conditions of Zn(II) depletion [191], and for high-affinity Zn(II) binding to ZigA [193]. It was recently discovered that, upon binding Mg(II) and GTP, CobW assembles a thiol-rich, metal-binding site that is likely localised to this same motif [156]. The site binds Co(II), and the Co(II) affinity for the GTP-complex is more than one thousand times stronger than for the GDP-complex (*K*_Co(II)_ ~ 30 pM *versus* ~100 nM, respectively). Notably, with reference to the availability of Co(II) in cells [93] (see Section 6.5), only the GTP-complex has a sufficiently tight affinity to acquire Co(II) *in vivo* [156].

Similarly to CbiK (Section 6.2), the metal affinities of CobW (in the GTP-bound ‘metal acquisition’ form) for Cu(I) and Zn(II) are tighter than for Co(II) [156]. *In vivo* Cu(I) binding can be ruled out, since intracellular Cu(I) availability in a cell is too low to enable its acquisition [93]. However, the remarkably high Zn(II) affinity (~190 fM) is sufficient for *in vivo* Zn(II) acquisition [156]. Since both Co(II) and Zn(II) bind at the same (or similar) sites, it was necessary to account for competition between these two metals to determine *in vivo* metal-occupancy of CobW. Thermodynamics favours Co(II) binding because the free energy difference between metal-binding to CobW and metal-binding to the cytosolic buffer (termed ΔΔG), is greater (*ie* more negative) for Co(II) than for Zn(II) [156]. Thus, Co(II) is the cognate metal.

#### 6.6.2 Allosteric coupling of nucleotide and metal binding

**Fig. 11:**
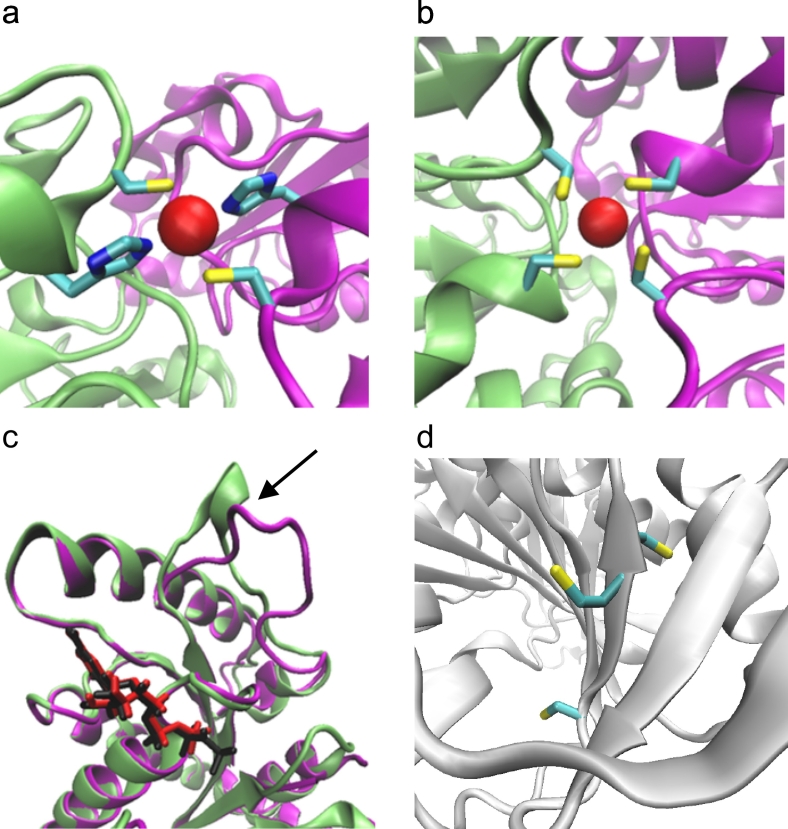
Allosteric relationship between nucleotide and substrate binding in G3E GTPases. a–b Square-planar Ni(II) coordination sites at dimeric interfaces in (a) *Klebsiella pneumoniae* UreG (in complex with GMPPNP; PDB 5XKT [220]) and (b) Helicobacter pylori HypB (in complex with GDP; PDB 4LPS [222]); the two protomers of each homodimer are coloured green and magenta, respectively. c) Structure of Methylorubrum extorquens MeaB in complex with GMPPNP (green; PDB 4JYB [226]) or GDP (magenta; PDB 4LC1 [226]); bound nucleotides are shown in black and red, respectively. Arrow points to the ‘Switch III’ loop, which changes conformation upon nucleotide hydrolysis and is crucial for chaperone function [226]. d) The CXCC putative metal-binding motif in *Escherichia coli* YjiA (of the COG0523 family; PDB 1NIJ [227]), crystallised in the absence of nucleotides.

In all G3E GTPases (Section 5.4.2.1) nucleotide binding and substrate binding are closely interconnected. UreG and HypB both form square-planar Ni(II) binding sites at dimerization interfaces which are induced by nucleotide binding (Fig. 11a,b) [[219], [220], [221]]. Moreover, the Ni(II) affinities of these sites vary significantly depending on the nature of the bound nucleotide (ie GDP *versus* GTP or a non-hydrolysable analogue) [[222], [223], [224]]. MeaB binding affinity for its substrate MCM is also affected by nucleotides [225]. X-ray crystallography has revealed nucleotide-dependent conformational changes in proteins from the MeaB [226], HypB [221,222] and UreG [219,220] families which rationalise these observations (Fig. 11a-c). However, structural determinations of CobW in complex with nucleotides are still needed to identify the configuration of the Co(II) site and to explain the substantial impact of nucleotide-binding on Co(II) affinity. Structural information is currently only available for one member of the COG0523 family (*E. coli* YjiA), and in the absence of bound nucleotides [192,227]. In these structures, the CxCC putative metal-binding motif is located along a beta strand in a conformation that would not likely support metal coordination (Fig. 11d). It is tantalising to note that this motif lies between the canonical switch I and switch II regions which change conformation as a function of GTP-binding and hydrolysis [196,228].

UV–visible spectroscopy of nucleotide-bound CobW is characteristic of tetrahedral Co(II) coordination geometry with three ligating thiols (originating, at least in part, from the CxCC motif) [156]. It is possible that GTP-binding induces a conformational change in CobW that imposes a tetrahedral geometry on the coordination site. This could explain why, in contrast to CbiK (and the Irving-Williams series generally, which applies in the absence of steric selection; see Section 6), CobW binds Co(II) more tightly than Ni(II) [156] because Ni(II) favours square planar coordination geometry (see Fig. 11a,b) due to ligand-field stabilisation for the d8 metal ion. Structural differences which alter the metal selectivity of different COG0523 proteins for Fe(II), Co(II) or Zn(II) (see Section 5.4.2.1) also remain to be discovered.

#### 6.6.3 Function and mismetalation

Thermodynamic calculations which established that CobW is specific for binding Co(II) *in vivo* (Section 6.6.1) assume ‘idealised’ cellular metal availabilities, where sensors are at the mid-points of their responses (Section 6.5.1). If metal availabilities diverge from idealised values, calculations of CobW metalation change. Indeed, when engineered B_12_-producing *E. coli* strains (which, notably, lack an identified dedicated cobalt import system [22]) are cultured in standard media, intracellular Co(II) availability is limited and mismetalation of CobW by Zn(II) is predicted [156]. Only upon Co(II) supplementation does the intracellular Co(II) availability increase enough to elicit a response from the Co(II) sensor RcnR and in turn to lead to predictions that Co(II) will displace Zn(II) from CobW.

*In vivo* studies show that CobW significantly enhances B_12_-production at limiting intracellular Co(II) availabilities and CobNST can only acquire Co(II) directly from the cellular milieu if intracellular Co(II) levels are high [156]. In common with the contributions of copper-metallochaperones to metal-supply [207], it is predicted that CobW is needed to deliver Co(II) for insertion into the corrin ring when intracellular buffering molecules are not metal-saturated but competing. Since GDP-bound CobW has a drastically weaker Co(II) affinity than the GTP-bound form, GTP-hydrolysis is predicted to trigger Co(II) release [156]. An appealing possibility is that CobNST may act as a guanine nucleotide activating protein (GAP) to target metal delivery to the chelatase complex. This would be consistent with mechanisms of related G3E GTPases: GTPase activity of MeaB is enhanced 100-fold in the presence of its protein partner MCM [225]; while enhanced (albeit to a lesser extent) NTPase activities of UreG and HypB have also been reported in the presence of their respective interaction partners UreE [224] and HypA [229]. It is not known whether the GTPase activity of CobW provides an additional ‘check-point’ for metal fidelity, or whether the chelatase CobNST, whose *in vitro* insertase activity appears remarkably specific for Co(II) [155], is solely responsible for metal selectivity at the insertion step.

## 7 Future prospects: food security, biotechnology and the metalation of vitamin B_12_

As increasing numbers of individuals adopt vegan or ‘flexitarian’ (with reduced consumption of animal products) diets, so B_12_ insufficiency becomes more prevalent. Complete synthesis of B_12_ is possible [230], but it is not an economically viable option for mass production. Native strains that make B_12_ are generally less well suited to large scale industrial fermentations than, for example, *E. coli* or yeast, making this the most expensive vitamin on the market with prices having risen significantly over the past few years in a volatile market. Commercial production of vitamin B_12_ is made more challenging by the need to add cobalt to the growth media and the need to dispose of this carefully after growth given the health and environmental concerns associated with the metal. Similar concerns are associated with the addition of cyanide during the extraction and purification of the nutrient. It is likely that changes associated with improvements to the safer production of vitamin B_12_ have been one of the drivers associated with the increase in price of the nutrient. Therefore, there exists an exciting prospect of improving B_12_ manufacture coordinated with precise cobalt addition by engineering the biosynthesis and uptake pathways into heterologous strains to produce cobalamin in a controlled manner to help facilitate a simpler purification.

Fig. 10a provides a thermodynamic framework which may be used to inform the optimisation of vitamin B_12_ metalation, either by manipulating media cobalt and/or competing ions (6.5.4, 6.6), or by engineering the metallostasis of cobalt and/or competing ions. More broadly, with half of the reactions of life requiring metals, this framework may be applied to the improved manufacture of a wide range of products through sustainable bio-transformations. However, this is also relevant to areas of human health, especially in the human gut microbiome, where around a third of bacteria produce cobamides that help sustain many of the other cobamide-dependent bacteria [231]. Corrinoids are known to play a key role as modulators of the microbiome and the availability and integration of cobalt into the corrin-framework is a key step in the health of this large and complex ecosystem [232]. Indeed, it is interesting to note that human cobalamin-deficiency is often associated with inflammatory bowel disease and bacterial dysbiosis [233].

Another area of developing interest is the synthesis of metal variants of cobalamin, where the central cobalt ion is replaced with an alternative transition metal ion. The properties of the different metal ions within the corrin ring means that most of these metal-corrin analogues tend to act as anti-vitamins. For instance, the inclusion of rhodium in place of cobalt produces rhodibalamin, which forms a stronger and more stable metal-carbon bond, which prevents catalytic turnover [16]. Replacement of cobalt with other metals such as zinc or nickel generate structures that more closely resemble the Co(II) or Co(I) corrins, respectively, and have significant potential in the study of B_12_-dependent processes [234,235]. Excitingly, the capacity to make metal-free forms of cobalamin linked with the ability to insert a range of metal isotopes also paves the way for the development cobalamin as an imaging agent [15].

Significant advances have been made from using cobalt and the corrin ring to gain new knowledge about how metal availability, binding and delivery are coordinated to produce metallo-products. There has been ongoing debate about the contribution of kinetic factors [208], mediated by specific protein interactions and metal-niches, to metalation. To what extent is metal supply ‘hard-wired’ from import to metalation *via* specific protein-interactions, or thermodynamically equilibrated with buffered intracellular pools. Probably some combination applies to differing degrees for different metalloenzymes and cofactors. The framework in Fig. 10a offers the exciting prospect of uncovering the answers to such questions.

## CRediT authorship contribution statement

**Deenah Osman:** Writing - review & editing. **Anastasia Cooke:** Writing - review & editing. **Tessa R. Young:** Writing - review & editing. **Evelyne Deery:** Writing - review & editing. **Nigel J. Robinson:** Conceptualization, Writing - review & editing. **Martin J. Warren:** Conceptualization, Writing - review & editing.

## Declaration of competing interest

The authors declare that they have no known competing financial interests or personal relationships that could have appeared to influence the work reported in this paper.
